# AI agents are sensitive to nudges

**DOI:** 10.1073/pnas.2537030123

**Published:** 2026-06-15

**Authors:** Manuel Cherep, Pattie Maes, Nikhil Singh

**Affiliations:** ^a^ Media Lab, Massachusetts Institute of Technology, Cambridge, MA 02139; ^b^ Department of Computer Science, Dartmouth College, Hanover, NH 03755

**Keywords:** agents, agentic AI, behavioral machine learning, alignment, safety

## Abstract

Large language models (LLMs) are increasingly deployed as autonomous agents that make choices and use tools on behalf of users. Yet, we have limited evidence about how their decisions are shaped by their environment. We adapt a human decision-making task to test leading LLMs under four forms of choice architecture: defaults, suggestions, information highlighting, and “optimal” nudges derived from a resource-rational model of human choice. We treat human behavior as a baseline for predictable sensitivity to such interventions. Across models and prompting strategies, LLMs often depart substantially from this baseline. They sometimes pay excessive costs to acquire information, sometimes ignore available information, and, most crucially, are far more responsive to nudges than humans, such that weak cues that slightly shift human behavior have larger effects on model choices, toward both better and worse payoff outcomes. Chain-of-thought prompting and in-context human data do not reliably stabilize behavior. Recent reasoning-optimized LLMs can, in some configurations, restore more human-level sensitivity to nudges, but do so inconsistently and at substantial computational cost. These results point to an important and largely neglected safety concern: LLM agents can be behaviorally brittle under subtle changes in choice architecture, even in the absence of adversarial settings.

## Significance

Many applications of AI agents tacitly assume that, under uncertainty, they will react in roughly human-like ways, if not more rationally. Using a benchmark designed to elicit human choice strategies, we show this assumption often fails. Rather, LLM-powered agents are typically much more sensitive to ordinary choice architecture cues. Because some are helpful and others are not, this can push model decisions toward higher or lower payoffs. This complements prior work on prompt robustness and adversarial attacks, because it is triggered by benign, semantically meaningful features of the decision environment. While frontier reasoning models partially mitigate these effects, this comes at a substantial cost. Our findings identify behavioral robustness as a fundamental challenge for safe and trustworthy AI agents.

We seem to want more from our language models than just a good conversation. Software agents (1) powered by LLMs can now in principle browse the web (2–4), use spreadsheets (5), go shopping (6), make financial decisions (7), and operate computer-based tools (8, 9). In such settings, they must make sequential decisions, often on behalf of people. Yet we know surprisingly little about how these systems actually choose. Do they follow human-like strategies? Or do they rely on very different heuristics that may be unstable, easily manipulated, or misaligned with human welfare?

Decades of research in behavioral science show that human decision-making is shaped not only by preferences but also by choice architecture (10, 11), i.e. the way options are presented. Theories of human cognition and behavior, such as bounded rationality (12), have shown how people deviate systematically from classical assumptions such as those made by expected utility theory (13, 14). Resource-rational models (15) formalize these constraints and provide quantitative accounts of when and how humans use cognitive resources to make decisions. Nudges such as defaults and suggestions exploit these tendencies to shift human choices in predictable ways. Whether LLM agents, increasingly used as proxies for human decisions, exhibit similar, different, or no sensitivities to such influences is unknown given that they do not share the computational limitations that humans do, which arise from biological constraints (16).

Recent work has shown that LLMs trained on human data do not reliably replicate human decision processes: They may model people as overly rational (17), misrepresent human trade-offs (18), amplify human biases (19), or diverge across models and prompting conditions (20–22). They have also been used to simulate human behavior in social and organizational contexts (20, 23–29), but these simulations rest on assumptions of alignment that may not hold. Moreover, LLMs are known to be fragile to small prompt or formatting changes (30–36), vulnerable to adversarial attacks (37, 38), and susceptible to sycophancy, i.e. overweighting a user’s stated views (39, 40). Our focus is different but complementary: Rather than arbitrary prompt perturbations or interpersonal agreement, we study semantically meaningful features of the task environment itself. LLMs have also very recently been shown to be susceptible to simple textual nudge-like statements in consumer choice tasks on realistic web platforms (41). Despite this, we have not had a precise way to characterize the nature and extent of such influences. Choice-architecture interventions such as defaults, suggestions, or highlighted information have interpretable directionality, known effects in humans, and measurable consequences for search costs and payoffs. This makes them a useful lens for asking whether model sensitivity is calibrated to the value of a cue or reflects indiscriminate compliance.

Here we directly compare humans and LLMs in a canonical multiattribute sequential decision-making task designed to probe responses to nudges (42). We test defaults, suggestions, information highlighting, and resource-rational “optimal” nudges across multiple frontier models and common prompting strategies. This stylized paradigm helps establish a controlled behavioral benchmark in which the direction and value of each intervention are interpretable. We find that LLMs not only deviate from human strategies but also often display hypersensitivity to nudges, responding more strongly than people (who are already sensitive to them), including in cases where the intervention is neutral or counterproductive. Alignment strategies such as chain-of-thought or few-shot prompting mitigate some baseline differences but do not eliminate hypersensitivity. A few state-of-the-art reasoning models, on the other hand, can achieve human-level sensitivity at a higher computational cost. Finally, we corroborate in *SI Appendix*, §3 that nudge sensitivity carries over to a real-data environment.

These findings expose a critical behavioral gap between human and model decision-making. They suggest the need for systematic frameworks to evaluate LLM choice processes and for safeguards when deploying agents in settings where small changes in the choice architecture could strongly bias outcomes.

## Results

### Task Paradigm and Interventions.

**Fig. 1.**
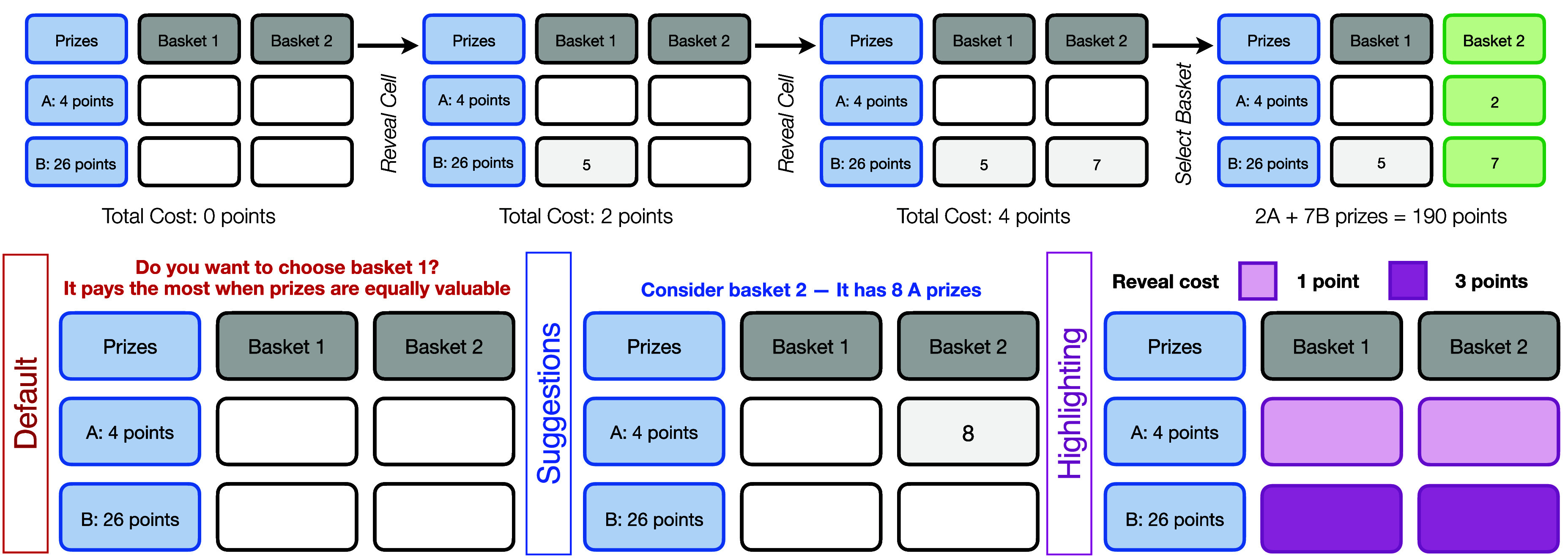
*Top*: Multiattribute sequential decision-making task adapted from ref. 42. Agents reveal cells at a cost and then choose a basket; net payoff equals the chosen basket’s reward minus reveal costs. *Bottom*: Three visually illustrated choice-architecture interventions (defaults, suggestions, and information highlighting). Optimal nudges, which we also study, use informative prereveals and are described further in the text.

To examine how LLM agents respond to choice architecture, we adapted a multiattribute tabular sequential decision-making task previously used to study human behavior (42) (Fig. 1). In this task, each trial presents a table of prize values in which rows correspond to prize categories and columns to baskets. Cell values are initially hidden. Agents may reveal cells one at a time, paying a cost for each reveal, and then select a basket. The reward is the weighted sum of the chosen basket’s prize values minus reveal costs, so good performance requires balancing the value of additional information against its cost.

We implemented the task as a text-based tool-use environment in which models could call functions to reveal cells and select baskets. We then compared LLMs and human participants across four nudge types: i) default options, ii) early and late suggestions, iii) information highlighting, and iv) resource-rational analysis-derived “optimal” nudges. These interventions make it possible to distinguish calibrated from indiscriminate responsiveness: Following a default can improve outcomes on average because the default is chosen by a simple heuristic; following a random suggestion is not inherently beneficial; revealing from the highlighted row is useful when that row is in fact most informative; and gains from optimal prereveals should reflect sensitivity to information value rather than indiscriminate compliance. We evaluated fourteen frontier models under base (task instructions only), zero-shot chain-of-thought (43, 44), and few-shot prompting with human examples.

### Nudge Sensitivity.

The central finding in this work is that most LLM agents are substantially more sensitive to nudges than humans across conditions, while increased thinking effort in state-of-the-art reasoning models can reduce such sensitivity. We analyzed choice outcomes with logistic regression models, with data source (human vs. different models), prompting method (base vs. chain-of-thought vs. few-shot human data) and trial condition (control vs. nudge, or optimal vs. suboptimal in the highlight condition) as fixed effects and cluster-robust SEs for participant. More detailed model specifications and estimates are given in *SI Appendix*, §6 and §7.

#### Default options.

**Fig. 2.**
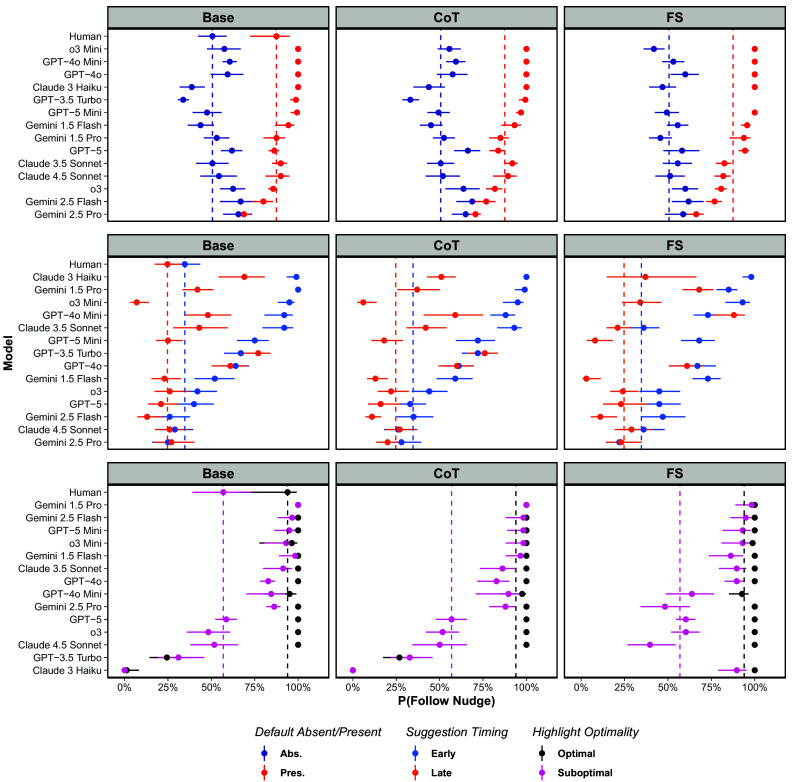
Probability of choosing the nudged basket or prize by model and prompting strategy (base, chain-of-thought, and few-shot human data) compared to human baselines (dashed lines).

Fig. 2 shows the probability of selecting the “default” option (without it being labeled as such). Because the default basket is chosen by a simple heuristic, accepting it can improve payoffs on average, but calibrated behavior should still depend on whether the basket is actually attractive. In control trials without defaults, humans chose the basket that would have been designated as the default 51% of the time on average. Most models came close to this baseline, except GPT-3.5 Turbo, which was significantly lower (34%, P<0.0001). With defaults present, humans increased to 88%. LLMs increased much more: GPT-3.5 Turbo (P=0.02) and GPT-5 Mini (P=0.03) both rose to 99%, Gemini 1.5 Flash to 94% (P=0.002), and GPT-4o, GPT-4o Mini, Claude 3 Haiku, and o3 Mini reached 100% acceptance, suggesting near-automatic compliance. Stronger reasoning models like Gemini 2.5 Flash, Gemini 2.5 Pro, Claude 4.5 Sonnet, o3, and GPT-5 were not significantly different from humans (all P>0.05).

#### Suggested alternatives.

Fig. 2 shows the probability of choosing the suggested basket in early (before initial choice) and late (after initial choice) conditions. Suggested baskets are random, so blanket acceptance and early-late differences are not generally optimal. In suggestion trials, humans accepted early suggestions 35% of the time. Models such as GPT-3.5 Turbo, GPT-4o Mini, GPT-4o, Gemini 1.5 Pro, Claude 3 Haiku, Claude 3.5 Sonnet, o3 Mini, and GPT-5 Mini exceeded this rate (all P<0.0001). In late suggestions, humans accepted 25%. Again, many models showed elevated acceptance (42 to 77%). Other models like Gemini 2.5 Flash (13%, P=0.03) and o3 Mini (7%, P=0.0006), which were inversely hypersensitive and dramatically reduced acceptance, indicating that the timing of the suggestion alone could strongly steer behavior even though timing does not change its value. In both suggestion timings, stronger reasoning models like o3, GPT-5, Claude 4.5 Sonnet, and Gemini 2.5 Pro were not significantly different from humans (all P>0.21).

#### Information highlighting.

We look at the probability that the first reveal came from the highlighted row in treatment trials only (Fig. 2). This behavior is optimal when the highlighted row has the highest prize, and therefore provides the most information. In optimal cases, humans made their first reveal from the highlighted row 94% of the time. However, GPT-3.5 Turbo (24%) and Claude 3 Haiku (1%) did not always reveal optimally. In suboptimal cases, humans reduced this rate to 57%, but most models overshot: Gemini 1.5 Pro (100%), Gemini 1.5 Flash (98%, P=0.0002), Gemini 2.5 Flash (97%, P<0.0001), Gemini 2.5 Pro (86%, P=0.0007), GPT-4o (83%, P=0.003), Claude 3.5 Sonnet (91%, P=0.003), o3 Mini (93%, P=0.002), and GPT-5 Mini (95%, P=0.001). Stronger reasoning models like o3, GPT-5, and Claude 4.5 Sonnet were not significantly different from humans (all P>0.6). In *SI Appendix*, §5, we also include results comparing the highlight-absent to highlight-present conditions.

#### Optimal nudging.

Finally, we test whether LLMs could benefit from optimized nudges: choice architectures derived from cognitive models of humans that are designed to maximize decision quality under cognitive constraints. In particular, we use nudges derived from a resource-rational model of human search (42). Unlike traditional nudge design, which is largely intuitive and domain-specific, this framework formalizes nudges as modifications to the meta-level decision problem, revealing initial values at the beginning of the trial. The central question here goes beyond whether models comply with a recommendation, but whether they make better use of higher-quality initial information. The framework predicts that nudges optimized in this way should outperform random or heuristic baselines.

These nudges specify which cells to reveal initially to maximize expected reward for humans. This gives a principled, out-of-distribution test for LLM agents: i) do nudges optimized under a human cognitive model transfer to LLMs (positive-control for value-sensitive information use), and ii) do any gains reflect adaptive integration of informative reveals rather than indiscriminate nudge-following? The design manipulates the quality of initial information while holding quantity fixed (six cells), enabling a graded test (Random → Extreme, i.e. three random and three most extreme abs. values → Optimal) that cleanly separates sensitivity to information value from generic nudge compliance.

**Fig. 3.**
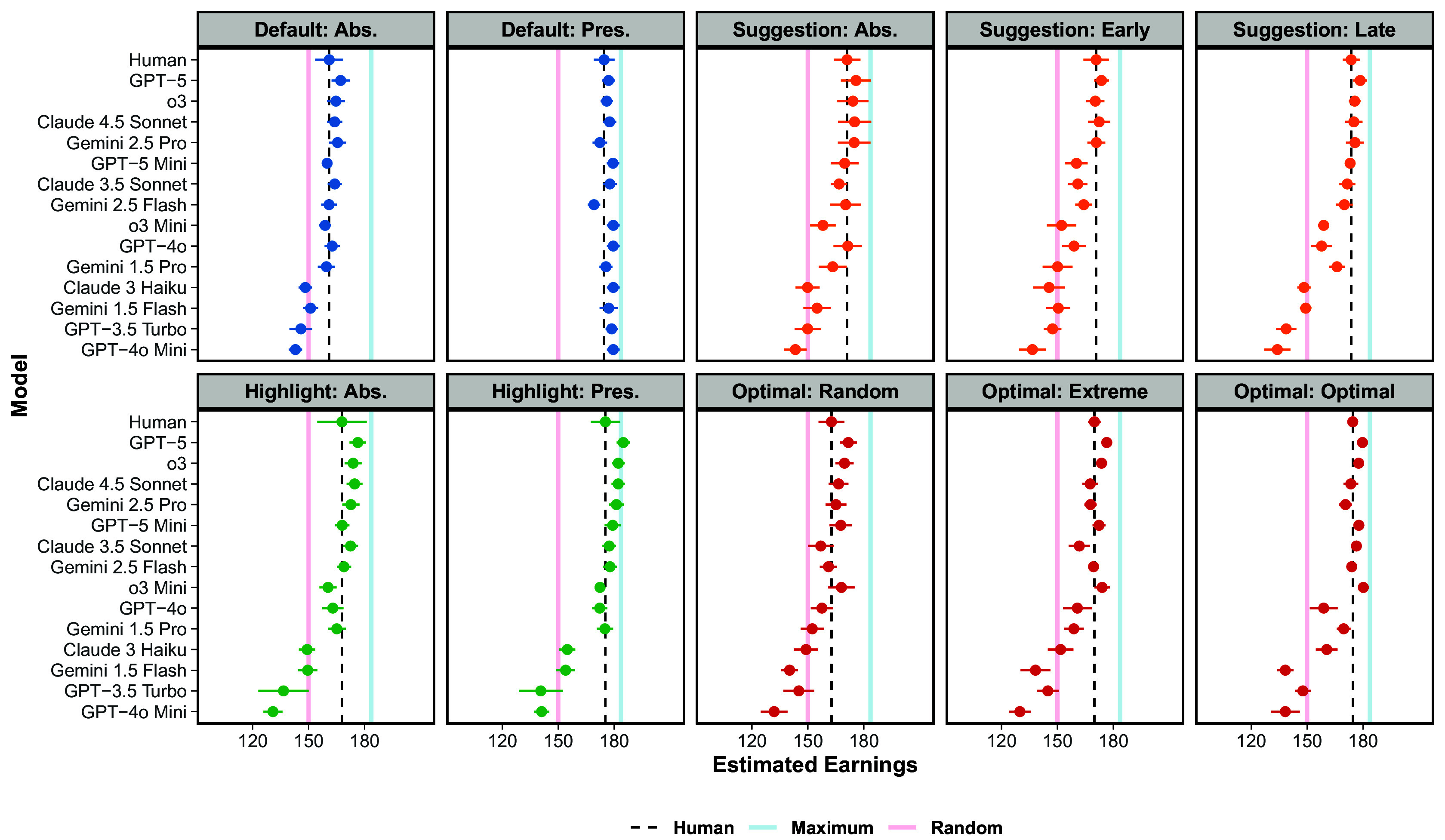
Net earnings per model in total points minus cost of revealing for all control and nudge conditions. The vertical lines represent the human payoff (dashed black), random payoff (red), and optimal payoff (blue). Comparing this figure with Figs. 2 and 4 shows that similar payoffs can arise from very different decision processes. The explicit change in earnings induced by nudges relative to a no-change baseline and relative to the human treatment effect can be seen more easily in *SI Appendix*, Fig. S8.

The original human data showed a clear monotonic increase (163 → 170 → 175 points; Fig. 3), consistent with the resource-rational analysis. Several models tracked this such as Gemini 1.5 Pro (152 → 159 → 170), Gemini 2.5 Flash (161 → 169 → 174), Gemini 2.5 Pro (165 → 168 → 171), Claude 3 Haiku (149 → 152 → 161), Claude 3.5 Sonnet (157 → 162 → 176), Claude 4.5 Sonnet (167 → 168 → 173), o3 Mini (168 → 174 → 180), o3 (170 → 174 → 178), GPT-5 (172 → 177 → 180), and GPT-5 Mini (168 → 172 → 178). Other models failed to improve with higher-quality reveals; e.g. GPT-4o Mini remained near 130 to 138 points across all policies (P<0.001 vs. humans), indicating potential insensitivity to information value. These heterogeneous effects suggest that higher payoffs under nudges need not signal strategy alignment: Some agents may accurately track information quality in a human-like way, while others may not. In *SI Appendix*, §4, we run additional experiments wherein we propose three distinct strategies to optimize nudges for LLM behavior from their observed trajectories, including a descriptive surrogate policy fitted to logged optimal-task trajectories, a direct supervised scorer neural network model from the fully observed task state, and a learned deviation from the resource-rational optimal nudge. Ultimately, we find that, for stronger reasoning models, the resource-rational optimal nudge is already well-suited to their behavior and it is difficult to discern enough empirical signal from the model’s observed behavior to exceed this.

### Information Acquisition Strategies.

To understand potential mechanisms underlying the observed nudge sensitivity, we compared how humans and LLMs acquire information before making a choice. In this task, each reveal incurs a cost, so optimal performance requires balancing the expected value of additional information against the cost of obtaining it. Human participants revealed a limited number of cells consistent with this tradeoff (42). LLMs, by contrast, often exhibited significantly divergent strategies.

**Fig. 4.**
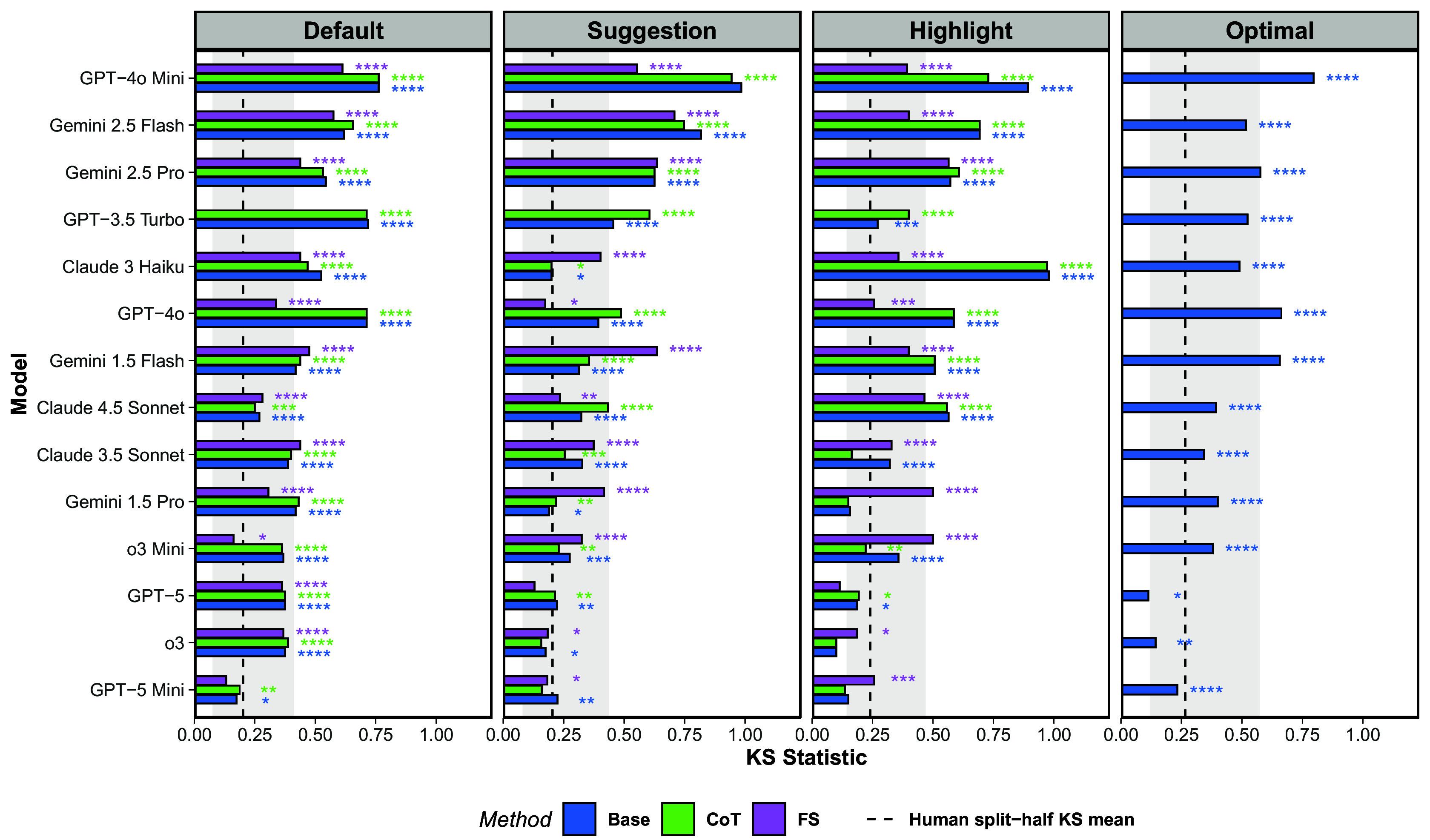
Kolmogorov–Smirnov (KS) test results comparing model reveal-count distributions to human participants’ by prompting strategy (base, chain-of-thought, and few-shot human data). Lower values indicate closer alignment; D=0 would indicate identical empirical distributions. ∗ shows a statistically significant difference (∗=P<0.05; ∗∗=P<0.01; ∗∗∗=P<0.001; and ∗∗∗∗=P<0.0001). The dashed line shows average human-vs-human KS distance from repeated participant-level split-halves within each experiment, as a benchmark range for how much KS variation arises when comparing one subset of humans to another. The shaded region shows empirical 95% interval from those split-halves (2.5th to 97.5th percentiles).

Fig. 4 shows the results of Kolmogorov–Smirnov (KS) tests, which confirmed significant differences between human and model distributions (see *SI Appendix*, §1, Fig. S1, for distribution plots) in nearly all cases (most P<0.0001, D=0.27 to 0.98). For example, GPT-3.5 Turbo rarely revealed at all, whereas GPT-4o Mini oversampled heavily, often uncovering entire rows or columns. Prompting strategies like zero-shot chain-of-thought reasoning and few-shot prompting with human examples had little effect. Saliency maps showing cell reveals (*SI Appendix*, §1, Fig. S2) pointed to structural biases: GPT-4o Mini frequently revealed in blocks and Gemini 1.5 Flash and Claude 3 Haiku favored leftmost columns or diagonals, for example.

### Net Payoffs.

Here, we examined net earnings (points earned minus information costs). This measure captures the bottom-line consequences of information acquisition strategies and nudge sensitivity (more in *SI Appendix*, §2). Importantly, this is equivalent to the typical evaluation metrics for AI agents, in that it reflects reward performance on the decision-making task. The main comparison in Fig. 3 is between outcome-level alignment here and process-level alignment in Figs. 2 and 4: Some models earn human-like rewards while remaining far more intervention-sensitive and strategy-divergent than humans. This also makes clear when nudge-sensitivity helps or hurts: Helpful defaults and optimal prereveals can improve payoffs, whereas random suggestions and suboptimal highlighting often do not. Here, we show how overreliance on this simple metric can therefore mask concerning misalignment and lead evaluators to falsely perceive alignment.

#### Default options.

In the control condition, humans earned 161 points on average. Model performance can be separated into four groups: underperformers (GPT-3.5 Turbo at 146, GPT-4o Mini at 143; both P<0.0001), moderately below (Gemini 1.5 Flash at 151, Claude 3 Haiku at 148; both P<0.0002), roughly human-level (GPT-4o at 163, Gemini 1.5 Pro at 160, Gemini 2.5 Flash at 161, Claude 3.5 Sonnet at 164, Claude 4.5 Sonnet at 164, o3-Mini at 159, and GPT-5 Mini at 160, Gemini 2.5 Pro at 166, o3 at 165), and above human-level (GPT-5 at 167, P=0.008). With a default present, humans improved to 175 points. Here, nearly all models also converged to 175 to 179 points, suggesting that they were aligning with human outcomes. However, as shown earlier, these apparent gains often reflect indiscriminate acceptance of defaults with a substantial base rate of nudge optimality, rather than selective information use.

#### Suggested alternatives.

In their baseline, humans earned 171 points on average. GPT-4o at 171, Gemini 1.5 Pro at 163, Gemini 2.5 Flash at 170, Gemini 2.5 Pro at 175, Claude 3.5 Sonnet at 167, Claude 4.5 Sonnet at 175, o3 at 174, GPT-5 at 176, and GPT-5 Mini at 170 closely matched this level, but others performed significantly worse (143 to 158). With early or late (random) suggestions, human payoffs remained similar (to 171 and 174). By contrast, most models remained below human levels (137 to 166), indicating suboptimal use of suggestion information despite their sensitivity to the nudges themselves. Only Gemini 2.5 Pro, Claude 4.5 Sonnet, o3, and GPT-5 continued to match human levels.

#### Information highlighting.

Without highlighting, humans averaged 168 points. Some models (GPT-4o at 163, Gemini 1.5 Pro at 165, Gemini 2.5 Flash at 169, Gemini 2.5 Pro at 173, Claude 3.5 Sonnet at 173, Claude 4.5 Sonnet at 175, o3 Mini at 160, o3 at 174, GPT-5 at 176, GPT-5 Mini at 168) were around this baseline, while others lagged (131 to 150). With highlighting, human earnings increased to 175 points on average. Some models achieved comparable payoffs, but others still remained far below (141 to 155).

### Acquisition Process vs. Net Payoff.

**Fig. 5.**
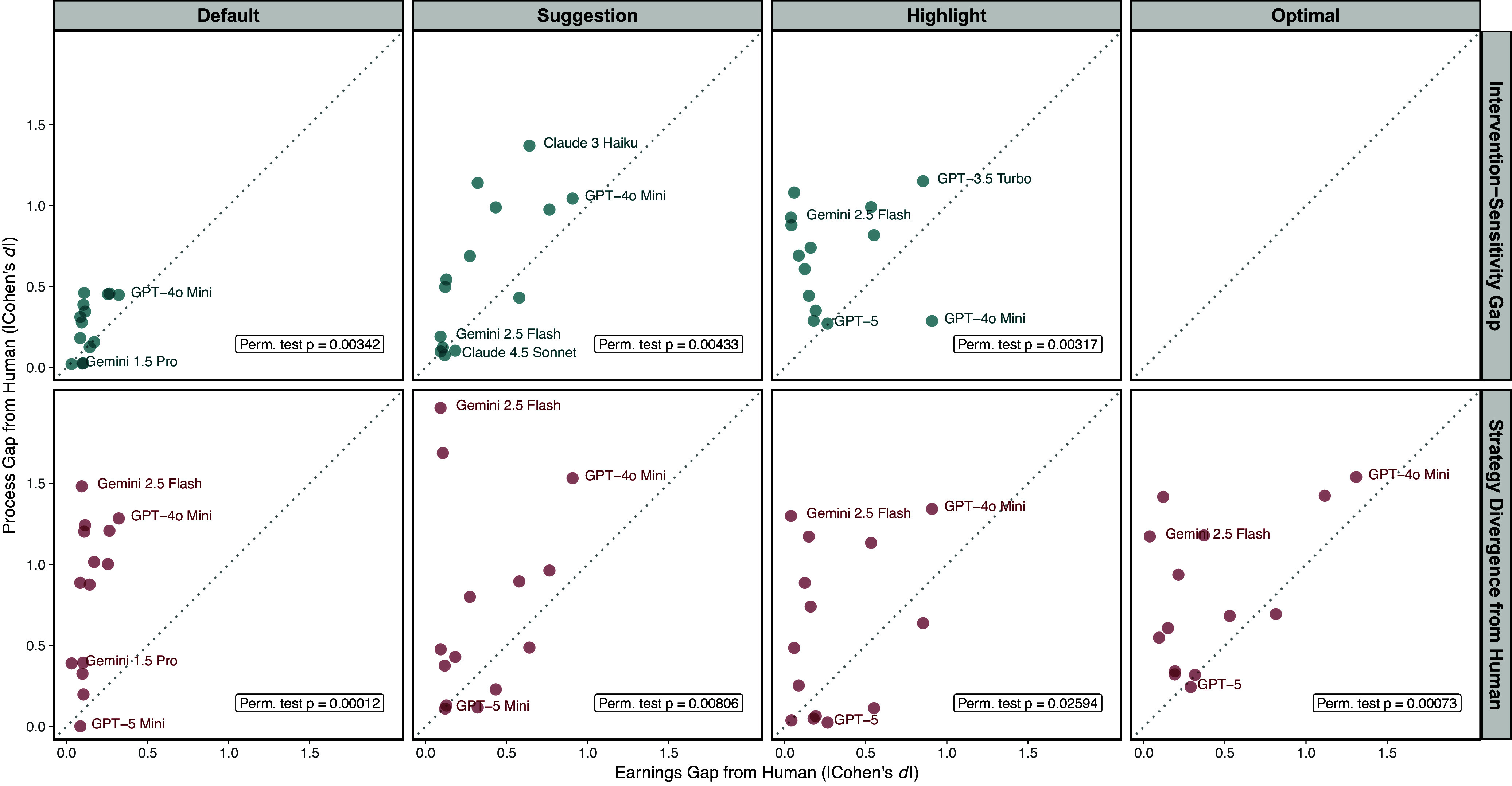
Absolute standardized earnings gap from humans plotted against absolute standardized process gap from humans. Process is measured either as intervention-sensitivity divergence or as strategy divergence. Points denote models, and the dotted diagonal marks equality between outcome and process divergence.

Fig. 5 compares each model’s absolute standardized gap (Cohen’s d) from humans in earnings to its absolute standardized gap from humans in process, where “process divergence” is measured either via intervention-sensitivity divergence or via strategy divergence. Process gaps frequently exceed outcome gaps, suggesting that models that look reasonably aligned on reward can still differ substantially in how they search for and use information. This pattern suggests that outcome metrics alone may understate subtle but potentially important process-level misalignment.

### Reasoning Effects.

Given the observed effect of reasoning (at the provider-default level in the main experiments), we analyze its influence at different levels of reasoning effort and its extra associated computational cost. We compare humans against GPT-5 (minimal, low, and medium), Gemini 2.5 Pro (low and medium), and Claude 4.5 Sonnet (low and medium). Note that minimal reasoning effort is not available for all providers, and that the reasoning levels are budgets and therefore not necessarily comparable across providers.

#### Probability of following intervention cues.

**Fig. 6.**
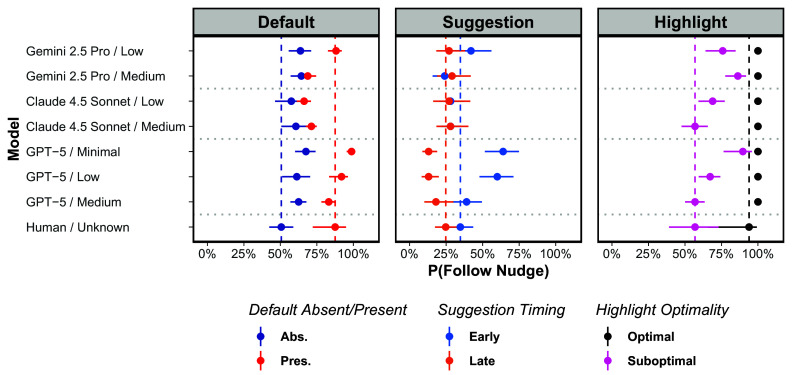
Probability of following intervention cues by model and reasoning effort level (minimal, low, and medium) compared to human baselines (dashed lines). Outcomes are default acceptance, choosing the suggested basket, and making the first reveal from the highlighted row.

Fig. 6 shows the probability of following intervention cues with defaults, suggestions, and information highlighting.

**Fig. 7.**
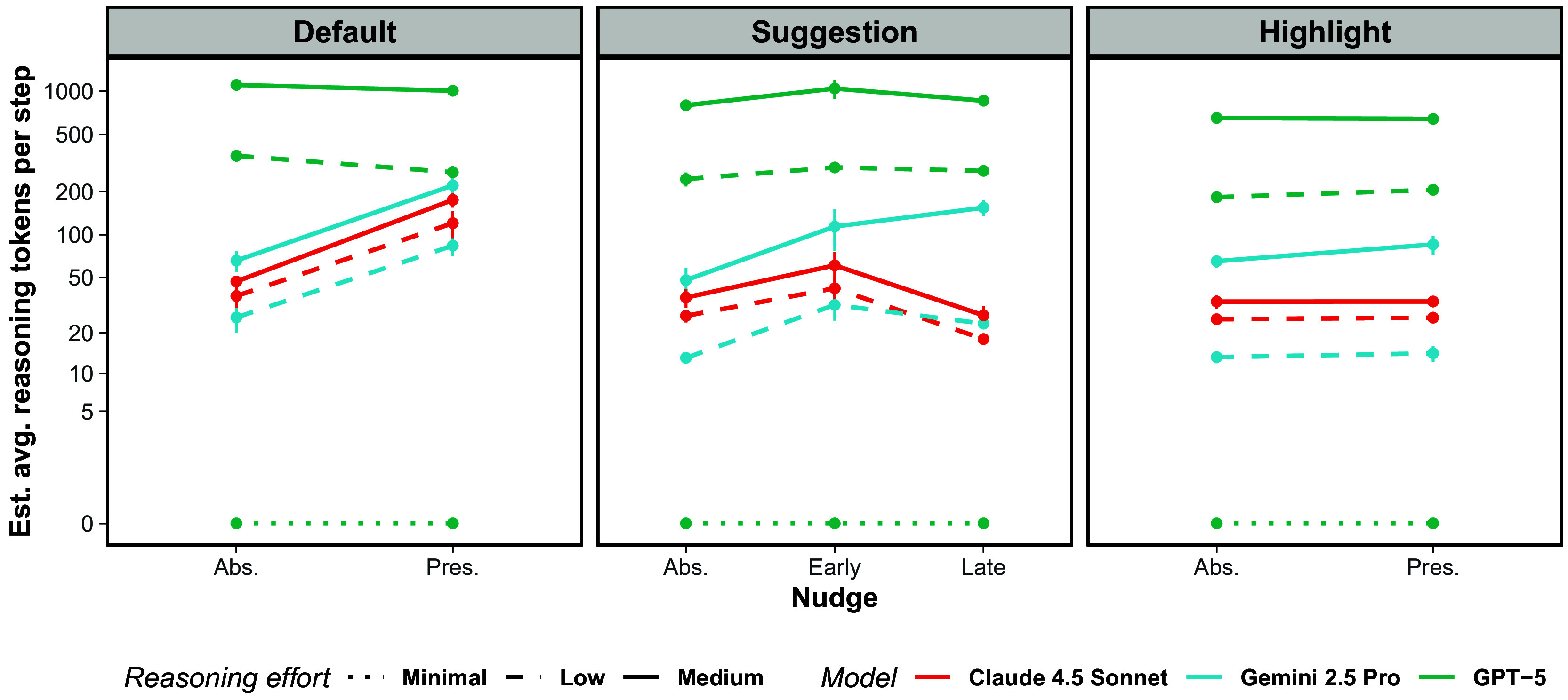
Estimated average reasoning tokens per step with different levels of reasoning effort (minimal, low, and medium) for GPT-5, Claude 4.5 Sonnet, and Gemini 2.5 Pro.
- **Suggestions**: In early suggestions, the probability of choosing the nudged basket decreases as the reasoning effort increases. Except Gemini 2.5 Pro Medium and Claude 4.5 Sonnet Low and Medium, all other models chose the early suggestions more often than people: GPT-5 Minimal at 64% (P=0.0005) and GPT-5 Low at 60% (P=0.001) were significant. In late suggestions, GPT-5 Minimal at 13% (P=0.01) and GPT-5 Low at 13% (P=0.01) were significantly different from humans. Overall, increasing reasoning effort reduces the behavioral gap between early and late suggestions to a human-like level.
- **Information Highlighting**: When the nudge is optimal, humans reveal cells from the nudged prize nearly universally (94%) and models reveal from it 100% of the time. However, when suboptimal, all models reveal from it more than humans do, which is significant for GPT-5 Minimal (P=0.01) and Gemini 2.5 Pro Medium (P=0.03). For GPT-5, the probability of choosing a suboptimal nudged prize decreases monotonically as reasoning effort increases (however, the inverse is true for Gemini 2.5 Pro’s two levels).

- **Default**: With defaults present, the probability of choosing the nudged basket decreases as reasoning effort increases. GPT-5 Minimal is the most sensitive at 99% (P=0.0007). Medium reasoning effort results in inverse sensitivity to the nudge, meaning that they all choose the nudged basket less than people do. Claude Sonnet 4.5 for both low and medium effort results in a very similar amount of reasoning (Fig. 7), thus the results are quite similar. Note that Claude models have a much higher minimum thinking budget than the others.

#### Cost of reasoning.

While increased reasoning effort can mitigate nudge sensitivity for the strongest frontier models, this robustness comes at substantial computational cost. Fig. 7 shows the average estimated reasoning tokens consumed per decision step across reasoning levels and models. The costs are considerable: Even at low reasoning effort for example, GPT-5 consumes ≈200 to 500 reasoning tokens per step. For context, a single trial in the default setting requires 1 to 12 decision steps.

In *SI Appendix*, §8, we estimate the monetary cost of these differences at current API pricing rates and show that, for GPT-5 in the default nudge for instance, this reflects a nearly 30× increase for low reasoning effort over the minimal effort baseline, and over ≈100× increase for medium. Even within the same nudge type and reasoning effort level, the effects can be substantial. For example, Gemini 2.5 Pro costs ≈3× as much with the default nudge present as without it. This may be due to additional reasoning effort needed to evaluate the nudge, for instance. For agentic applications involving thousands of daily decisions, these costs could amount to hundreds or thousands of dollars monthly, solely to achieve human-level behavioral robustness (which we emphasize is still sensitive to nudges). Conversely, resource-constrained deployments may be more vulnerable to nudges.

## Discussion

These results reveal a systematic divergence between humans and LLMs in sequential decision-making tasks shaped by choice architecture, such as the tasks routinely carried out by modern web agents (3, 6). The core contribution of these results, as compared with prior work on sensitivity to arbitrary input details [like punctuation, formatting, prompt wording, or adversarial changes (30–33, 35–38)], is that here the perturbations are semantically meaningful, theory-motivated features of the decision environment: defaults, recommendations, salience manipulations, and informative prereveals. This lets us ask whether observed behavioral changes are calibrated to the value of the cue, comparable to human behavior, and beneficial or harmful in payoff terms.

The behavioral strategies used by these agents point to unstable and idiosyncratic information acquisition. Some models terminated decisions without acquiring any information; others revealed excessively at high cost, sometimes uncovering entire rows or columns. Few-shot prompting reduced these extremes in some cases by encouraging more human-like sampling, but ultimately did little to eliminate the nudge-sensitivity. This suggests that without models learning more principled strategies for information acquisition, even mild changes in choice architecture can dominate model behavior, making them vulnerable to manipulative or accidental nudges. Supporting this, we observe human sensitivity levels in state-of-the-art models when given a sufficient reasoning budget. Nonetheless, this extra reasoning comes at a high computational cost.

These behavioral instabilities also challenge standard evaluation practices. In AI benchmarking, rewards (here, net payoffs) are the primary measure of success. Yet our findings show how payoffs can be misleading: Models sometimes matched or exceeded human earnings under nudges, but some of these apparent successes were driven by hypersensitivity and indiscriminate compliance, behaviors that are far from ideal. Apparent gains in outcomes therefore masked deep misalignment in the underlying processes. This exposes a fundamental evaluation pitfall: Reliance on payoffs alone risks false confidence in AI alignment.

This phenomenon is also related to, but distinct from, sycophancy (39, 40). In sycophancy, models are swayed by a user’s stated beliefs or preferences. Here, the steering signal is embedded in the task environment or interface rather than in an interlocutor’s opinion. There is an overlapping concern regarding models’ overreadiness to treat contextual signals as instructions, but choice architecture studied this way allows for a more precise distinction between helpful, neutral, and harmful influences.

Our study also has an important limitation. The task is deliberately controlled and stylized, which gives strong causal interpretability but does not by itself establish how large these effects will be in every realistic deployment setting. Recent evidence from ABxLab (41) suggests that nudge-like effects can be large in a realistic web environment, in some cases. We therefore view the present results as evidence for a safety-relevant behavioral mechanism, and urge the research and practitioner community to test similar interventions in a variety of richer agent environments.

Taken together, these results highlight both risks and opportunities. The risk is clear: When deployed as agents, LLMs may be systematically biased by minor features of their digital environments, leading to fragile or manipulable behavior. The opportunity is that frameworks like this from cognitive and behavioral science provide principled tools for probing and potentially improving AI decision-making. Behavioral tests of the kind we introduce here can serve as stress tests for alignment, exposing hidden vulnerabilities that outcome metrics obscure, and providing systematic ways to compare and improve models before deployment.

Ultimately, these findings suggest that aligning LLM agents with human decision-making cannot rely solely on optimizing outcomes or drawing on human data at inference-time. It requires deeper behavioral evaluation frameworks that test for robustness to choice architecture, sensitivity to information value, and stability of search strategies. Developing such frameworks is not only necessary for safe and trustworthy deployment, but also contributes to the broader science of decision-making by clarifying how human and artificial agents diverge under uncertainty.

## Materials and Methods

### Task Paradigm.

We adapted a canonical multiattribute sequential decision-making paradigm developed for human behavioral studies (42). In this task, participants (or agents) choose among baskets of prizes with partially hidden values (Fig. 1). Agents may reveal these values one cell at a time, with a cost depending on the nudge, before selecting a basket. The reward is given by the sum of revealed prize values in the chosen basket. The objective is to maximize reward while minimizing information costs (i.e. to maximize net reward). Human data show that behavior in this task is well captured by resource-rational models (15) of bounded decision-making.

### LLM Adaptation.

We converted the game into a text-based format suitable for large language models (LLMs). The grid was presented as a Markdown table with hidden cells indicated by “?”. LLMs could interact with the game through callable functions for reveal (to uncover a cell), select (to choose a basket), or accept/decline (in default trials). Instructions were minimally rephrased into natural language while preserving the structure of the task. Preliminary tests of different tabular encodings (CSV, XML, HTML) suggested that Markdown yielded the most reliable model behavior, consistent with prior work on table formatting (34). Because the nudges were implemented purely textually, no visual input modality was required.

### Models and Prompting Conditions.

We evaluated fourteen state-of-the-art LLMs with function-calling capabilities: GPT-3.5 Turbo, GPT-4o Mini, GPT-4o, o3 Mini, o3, GPT-5 Mini, GPT-5 (OpenAI); Claude 3 Haiku, Claude 3.5 Sonnet, and Claude 4.5 Sonnet (Anthropic); and Gemini 1.5 Pro, Gemini 1.5 Flash, Gemini 2.5 Pro, and Gemini 2.5 Flash (Google). All models were run with a temperature of 0.2 if available, or the default behavior otherwise. To examine robustness, we tested three prompting conditions: i) **Base**, with only task instructions; ii) **Chain-of-thought (CoT)**, with zero-shot reasoning prompts (43, 44); and iii) **Few-shot**, with demonstrations drawn from human participants, balanced across control and nudge conditions. In total, experiments consumed roughly two billion tokens across models (inputs and outputs).

### Nudge Manipulations.

We implemented the four families of choice architecture manipulations from prior work (42):

1. **Default options**: Defaults were chosen as the basket with the most unweighted prize entries (thus, this is most clearly optimal when the prizes are all equal, since it becomes equivalent to the unweighted case). In nudge trials, the default basket could be accepted or declined at the start. In control trials, agents were not nudged to choose the default basket. The cost of revealing is 2 points per cell. The task is available online.
2. **Suggested alternatives**: A random basket was suggested either at the start of a trial (early suggestion) or an extra basket was added after a provisional choice (late suggestion). One high-value prize cell in the suggested basket was revealed for free. The cost of revealing is 2 points per cell. The task is available online.
3. **Information highlighting**: The cost of revealing cells from one randomly chosen prize row was changed (1 point vs. 3 points for others), incentivizing a more focused exploration strategy. In control trials, the cost of revealing is 3 points for all prizes. The task is available online.
4. **Optimal nudges**: Six cells were prerevealed at the start of a trial, choosing three randomly. The locations of the other three were chosen either randomly, by extreme values (furthest from 5 in either direction), or according to a resource-rational optimization model designed to maximize human performance (42). The task is available online.

In each case, an equivalent number of control trials without nudges were included.

### Reasoning Levels.

The strong reasoning models were tested at different levels of increased reasoning but, due to the providers, these levels are not necessarily comparable across models. Note that these are budgets, and the reasoning tokens used in practice can vary.

- **GPT-5**: This model has the following predefined reasoning efforts: minimal, low, medium, and high. However, the exact tokens are not documented.
- **Gemini 2.5 Pro**: The thinking budget ranges from 128 to 32,768 tokens. We use reasoning effort low (128) and medium (1,024). We do not use minimal, since this parameter cannot be zero.
- **Claude 4.5 Sonnet**: The minimum extended thinking budget is 1,024 tokens, and we set that as the low reasoning effort. The medium effort is 8,192 tokens, which is proportional to Gemini’s effort increase (8×). We do not use minimal, since this parameter cannot be zero.

### Trial Structure and Sampling.

The human baseline experiments consisted of a quiz, practice rounds, and multiple scored test games. We matched these designs closely, including identical prize distributions, cost structures, and basket configurations. For each model and prompting condition, we sampled approximately 300 to 340 trials per nudge type (340 for defaults, 320 for suggestions, and 300 each for highlighting and optimal nudges). Few-shot prompts included 12 human-played examples (balanced across conditions) where, in each one, at least one reveal occurred. Examples were sampled from unseen trials (i.e. different values compared to any of the games played by the AI agents), to avoid information leakage into the agents’ strategies.

### Statistical Analysis.

We analyzed model behavior along three dimensions: i) information acquisition strategies, ii) choice outcomes (nudge sensitivity), and iii) net earnings (reward minus reveal costs). All tests compared LLMs to human benchmark data from ref. 42, matched on trial structures. Reported P-values were adjusted using the Benjamini–Hochberg procedure within families (tests from the same regression model).

#### Information acquisition.

To compare the distribution of cells revealed before making a choice, we used two-sample Kolmogorov–Smirnov (KS) tests between each model–prompting condition and human participants. The KS D statistic (0–1) summarizes the maximum difference between empirical cumulative distributions and was used as a scalar measure of alignment. This test allows us to compare full empirical reveal-count distributions without assuming a particular parametric form. Though the counts are discrete here, the impact is limited given the sample sizes used here and, if anything, typically leads to more conservative *P*-values. As a robustness check, we re-estimate the *P*-values using Monte Carlo simulation in *SI Appendix*, Fig. S3, and the substantive conclusions remain similar.

#### Nudge sensitivity.

To test whether LLMs were more sensitive to nudges than humans, we fit logistic regression models predicting binary outcomes (e.g. whether a default was accepted, whether a suggested basket was chosen, or whether the first reveal came from the highlighted row). We used cluster-robust SEs on participant ID (inherited from the original human trials). For the models, the IDs serve as a conservative adjustment for within-session correlations: Each session (indicated by participant ID) involves session context material such as initial practice trials, thus violating independence. Fixed effects included data source (human vs. different models), prompting method (base, chain-of-thought, or few-shot human data), trial condition (control vs. nudge, optimal vs. suboptimal, early vs. late), and all interactions. We favored a saturated model specification to derive estimated marginal means from. Our test results are based on post hoc contrasts between model-estimated marginal means of human vs. LLM nudge sensitivity rates.

#### Net earnings.

To assess the bottom-line consequences of different strategies, we fit linear regression models with the same cluster-robust SEs as before, predicting total net points earned. Fixed effects included data source, trial condition (here, always nudge absence/presence conditions), prompting method, and their interactions. We reported model-estimated marginal means alongside post hoc contrasts vs. estimated marginal mean human earnings. For reference, the random baseline payoff is 150 points and the resource-rational optimum is ≈183.64 points.

### Disclosure of Delegation to Generative AI.

The authors declare the use of generative AI in the research and writing process. According to the GAIDeT taxonomy (2025), the following tasks were delegated to GAI tools under full human supervision: Code generation, Code optimization, and Proofreading and editing.

The GAI tool used was Claude 4.5 Sonnet, GPT-5 Codex. Responsibility for the final manuscript lies entirely with the authors. GAI tools are not listed as authors and do not bear responsibility for the final outcomes. Declaration submitted by: Authors.

## Supplementary Material

Appendix 01 (PDF)

## Data Availability

Tabular data in CSV format have been deposited in GitHub (https://github.com/PapayaResearch/nudging). Previously published data were used for this work (42).
