# Supplementary material for: AI agents are sensitive to nudges
Source: Proc Natl Acad Sci U S A. 2026 Jun 15;123(25):e2537030123. doi: 10.1073/pnas.2537030123 (PMC13291645; doi:10.1073/pnas.2537030123)
Supplement: Supplementary file 1 — Appendix 01 (PDF) [file pnas.2537030123.sapp.pdf]

PNAS

Supporting Information for

AI Agents are Sensitive to Nudges

Manuel Cherep, Pattie Maes, Nikhil Singh

Manuel Cherep.
E-mail: mcherep@mit.edu

This PDF file includes:

Supporting text
Figs. S1 to S11
Tables S1 to S18
SI References

Supporting Information Text

1. Distribution of Reveal Counts

A key behavioral signature we observe in the multi-attribute decision-making task is the number of cells agents choose to reveal before making a selection. This metric captures the information acquisition strategy, i.e. too few reveals suggest underutilization of available information, while excessive reveals indicate inefficient use of costly resources. Figure S1 displays kernel density estimates of reveal counts across all models, prompting strategies, and experimental conditions.

Human participants exhibit a reveal distribution biased toward fewer reveals, reflecting a balanced approach that weighs information value against acquisition costs. In contrast, LLM agents typically show notably different patterns. GPT-3.5 Turbo rarely reveals any cells, often making selections based solely on initial conditions (or arbitrarily). This strategy, while minimizing costs, forgoes potentially decision-relevant information. At the other extreme, GPT-4o Mini frequently reveals 8–12 or more cells, sometimes uncovering entire rows or columns. This oversampling behavior incurs substantial costs that can outweigh gains from additional information.

Prompting strategies have limited impact on these fundamental patterns. Chain-of-thought prompting produces modest shifts toward human-like distributions for certain models but does not fundamentally restructure the information acquisition strategy. Few-shot prompting with human demonstrations shows somewhat stronger effects in certain cases. Nevertheless, significant distributional differences generally across all conditions.

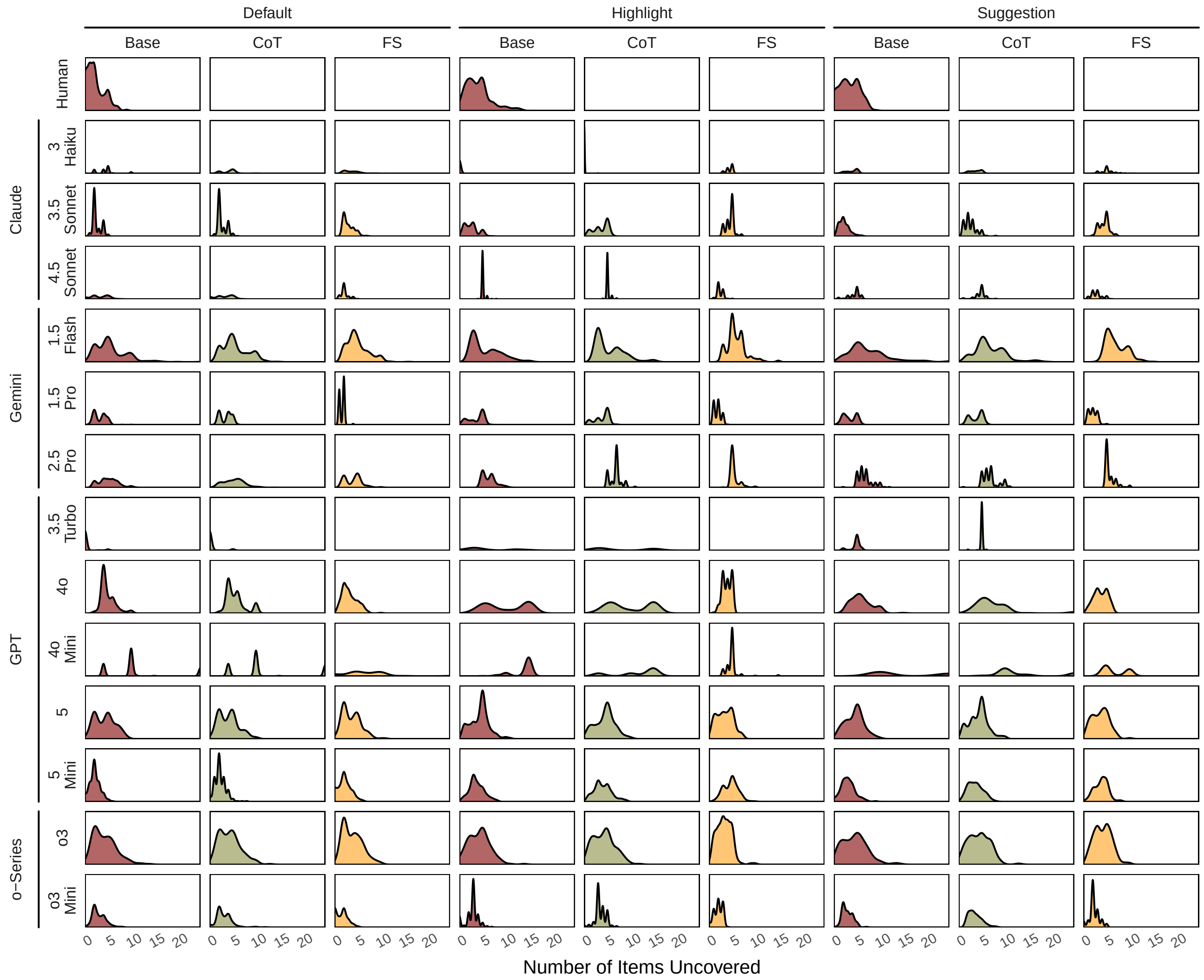

Fig. S1. Density plot showing distributions of reveal counts across trials, models, prompting strategies, and experiments.

Beyond the quantity of reveals, the *spatial structure* of information acquisition offers further insight into the heuristics guiding agent behavior. Figure S2 presents heatmaps showing cell reveal frequencies across the grid structure for each model and experimental condition (control conditions only, from the default nudge experiments). These visualizations expose certain systematic biases in how agents explore the decision space.

Human participants show relatively uniform sampling across cells. Some LLMs, however, exhibit pronounced spatial biases. For example, they may be biased towards left-side columns (Claude 3 Haiku, GPT-4o Mini to some extent), center columns (Claude 3.5 Sonnet, Gemini 1.5 Pro, GPT-5 Mini to some extent), the diagonal (Claude 3 Haiku, more pronounced with few-shot human data), and more. Such positional heuristics like column-wise and diagonal scanning strategies are unlikely to be well-aligned with with value-driven exploration (for example, revealing from the highest-prize row).

Such spatial biases may also have practical implications for deployment. An agent that systematically favors certain positions could be predictably manipulated by adversaries who understand these tendencies, or may consistently miss valuable information located in disfavored regions of the choice space.

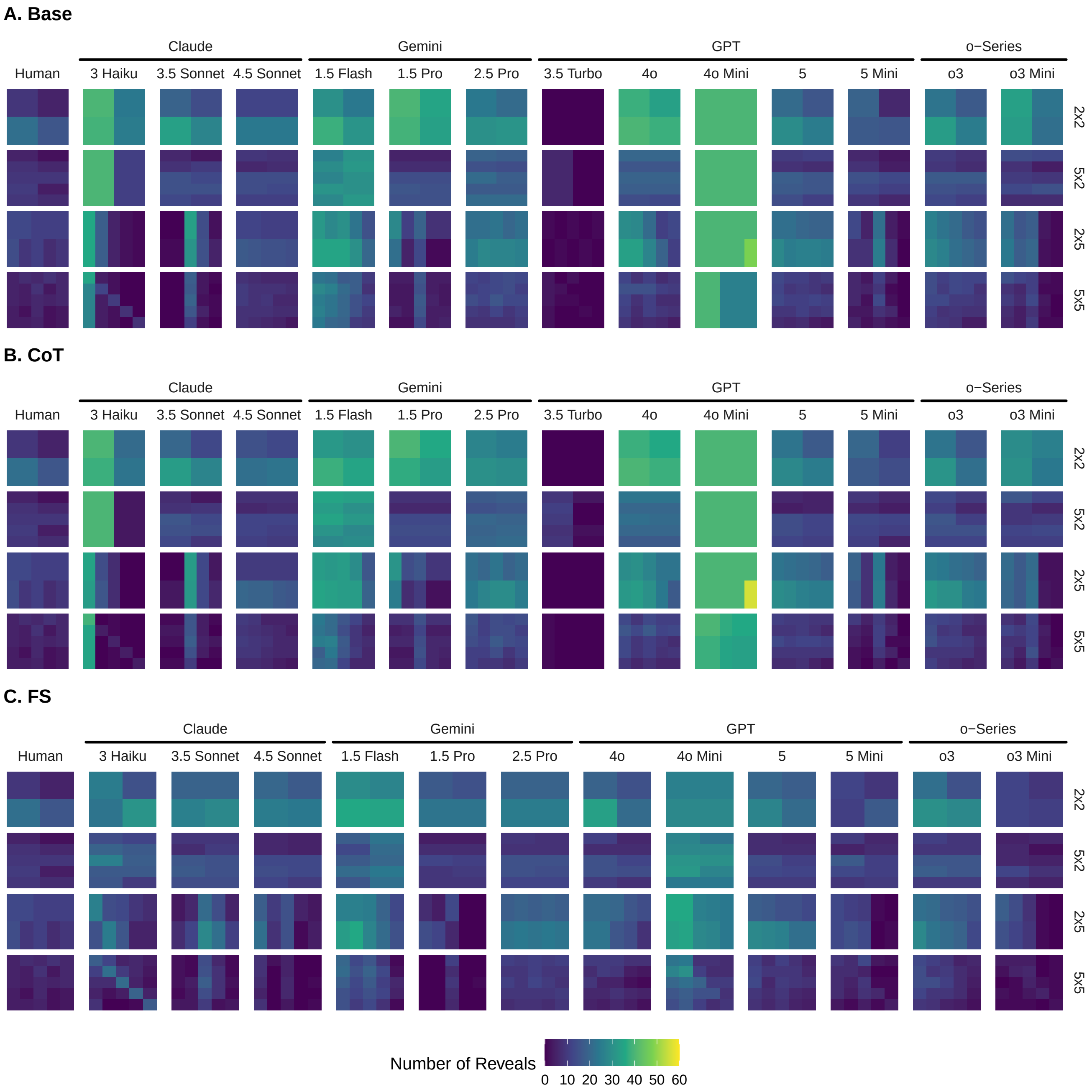

Fig. S2. Heatmap showing reveal counts across the spatial structure of each problem type (2 or 5 rows or columns).

Table S1 presents complete KS statistics and p-values for all model–prompting combinations, as shown in Figure 3 in the main text. These results suggest that current LLMs, regardless of prompting strategy, do not naturally or reliably replicate human information acquisition patterns. This behavioral divergence may be one factor contributing to the sensitivity to nudges

documented in the main text: agents that do not engage in balanced information search strategies may be more susceptible to environmental cues that substitute for self-directed exploration.

Table S1. KS statistics across all models and experiments.

		KS Statistic			
source	method	Default	Suggestion	Highlight	Optimal
GPT-3.5 Turbo	Base	0.72****	0.46****	0.27***	0.52****
GPT-3.5 Turbo	CoT	0.71****	0.61****	0.4****	NA
GPT-4o Mini	Base	0.76****	0.98****	0.89****	0.8****
GPT-4o Mini	CoT	0.76****	0.94****	0.73****	NA
GPT-4o Mini	FS	0.61****	0.55****	0.39****	NA
GPT-4o	Base	0.71****	0.39****	0.59****	0.66****
GPT-4o	CoT	0.71****	0.49****	0.59****	NA
GPT-4o	FS	0.34****	0.17*	0.26***	NA
Gemini 1.5 Flash	Base	0.42****	0.31****	0.51****	0.66****
Gemini 1.5 Flash	CoT	0.44****	0.36****	0.51****	NA
Gemini 1.5 Flash	FS	0.47****	0.64****	0.4****	NA
Gemini 1.5 Pro	Base	0.42****	0.19*	0.16	0.4****
Gemini 1.5 Pro	CoT	0.43****	0.22**	0.15	NA
Gemini 1.5 Pro	FS	0.31****	0.42****	0.5****	NA
Gemini 2.5 Flash	Base	0.62****	0.82****	0.69****	0.52****
Gemini 2.5 Flash	CoT	0.66****	0.75****	0.69****	NA
Gemini 2.5 Flash	FS	0.57****	0.71****	0.4****	NA
Gemini 2.5 Pro	Base	0.54****	0.63****	0.57****	0.58****
Gemini 2.5 Pro	CoT	0.53****	0.63****	0.61****	NA
Gemini 2.5 Pro	FS	0.44****	0.64****	0.56****	NA
Claude 3 Haiku	Base	0.52****	0.2*	0.98****	0.49****
Claude 3 Haiku	CoT	0.47****	0.2*	0.97****	NA
Claude 3 Haiku	FS	0.44****	0.4****	0.36****	NA
Claude 3.5 Sonnet	Base	0.39****	0.33****	0.32****	0.34****
Claude 3.5 Sonnet	CoT	0.4****	0.25***	0.16	NA
Claude 3.5 Sonnet	FS	0.44****	0.37****	0.33****	NA
Claude 4.5 Sonnet	Base	0.27****	0.32****	0.56****	0.39****
Claude 4.5 Sonnet	CoT	0.25***	0.43****	0.56****	NA
Claude 4.5 Sonnet	FS	0.28****	0.23**	0.46****	NA
o3 Mini	Base	0.37****	0.27***	0.36****	0.38****
o3 Mini	CoT	0.36****	0.23**	0.22**	NA
o3 Mini	FS	0.16*	0.32****	0.5****	NA
o3	Base	0.37****	0.18*	0.1	0.14**
o3	CoT	0.39****	0.16	0.1	NA
o3	FS	0.37****	0.18*	0.19*	NA
GPT-5	Base	0.37****	0.22**	0.19*	0.11*
GPT-5	CoT	0.37****	0.21**	0.19*	NA
GPT-5	FS	0.36****	0.13	0.11	NA
GPT-5 Mini	Base	0.18*	0.22**	0.15	0.23****
GPT-5 Mini	CoT	0.19**	0.16	0.14	NA
GPT-5 Mini	FS	0.13	0.18*	0.26***	NA

Given the discrete setting, we also repeated the analysis with Monte Carlo simulated p-values as a robustness check. Figure S3 shows that the qualitative pattern and overall conclusions are not meaningfully changed, i.e. the process-divergence conclusion does not hinge on the asymptotic KS approximation.

2. Earnings Effects and Process–Outcome Alignment Measures

To make the outcome consequences of nudges more explicit, Figure S4 plots the change in earnings induced by each nudge relative to two baselines: zero change and the corresponding human treatment effect. This view complements the acceptance-rate analyses above by distinguishing compliance with a nudge from potentially useful compliance. When the nudge is informative, stronger models can sometimes match or even exceed the human earnings gain, whereas weaker models often fail to extract comparable value. When the nudge is not guaranteed to improve outcomes, more models underperform compared to the human earnings difference, indicating that heightened sensitivity often doesn't translate into good decisions.

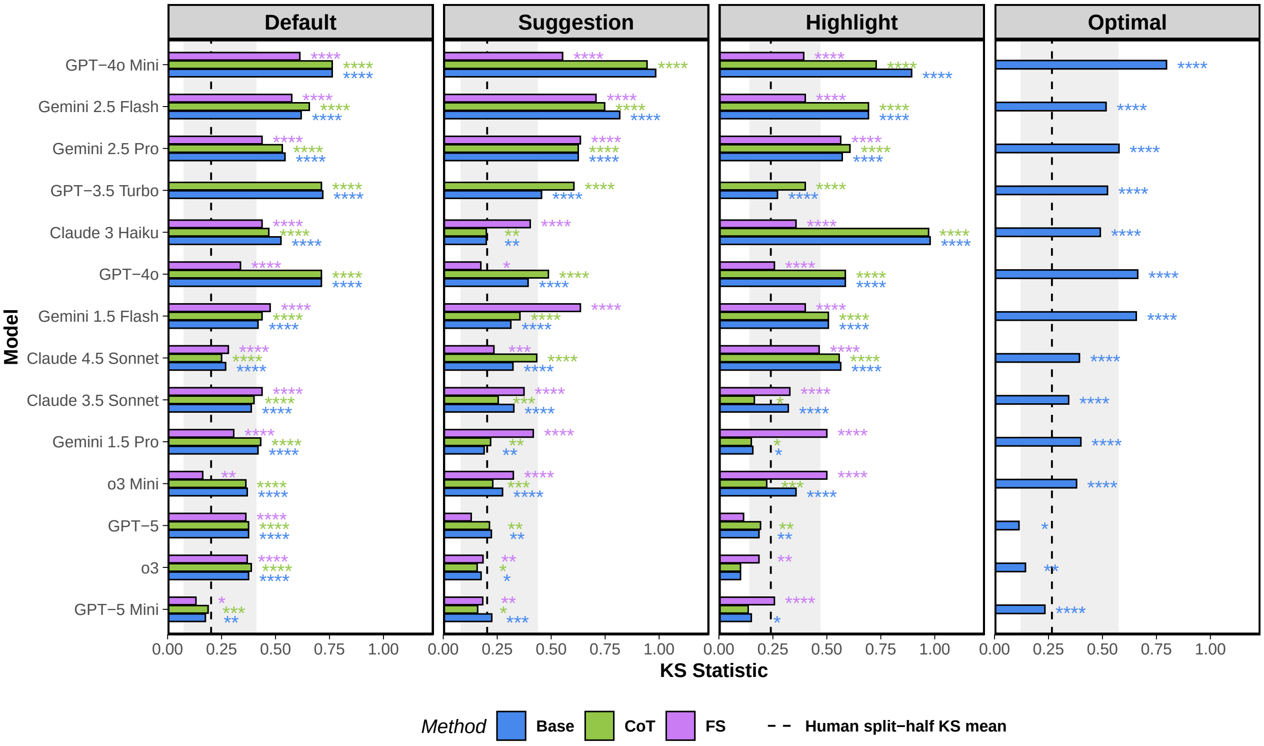

Fig. S3. Robustness check for the KS comparisons in Figure 3 of the main text, recomputing significance with Monte Carlo simulated p-values rather than the default asymptotic approximation. The qualitative pattern is unchanged. Dashed line shows average human-vs-human KS distance from repeated participant-level split-halves within each experiment, as a benchmark range for how much KS variation arises when comparing one subset of humans to another. Shaded region shows empirical 95% interval from those split-halves (2.5th to 97.5th percentiles).

3. Replication with Realistic Semantics

To bridge the stylized grid task and more realistic decision settings, we repeated the nudge manipulations in a selection experiment built from real housing data (1). The task preserves the same information-acquisition structure as the main experiments, but the options and attributes now correspond to semantically meaningful features. Figure S5 reports model-specific marginal probabilities of nudge following (similar to Figure 2 in the main text) in this setting.

The same broad qualitative pattern reappears here. Present defaults sharply increase choice of the default option. Suggestions are followed much more often when delivered early than late despite no meaningful differences, though late suggestions can still move behavior. Highlights are more selective: models are much more likely to act on optimal than suboptimal highlights, but several still show nontrivial attraction to suboptimal highlighted options. These effects are summarized as treatment contrasts in Figure S6. Together, these results support the claim that the phenomena documented in the main task persist when the decision problem is grounded in a realistic domain.

4. Optimized Nudges

We additionally evaluated whether nudges optimized *directly* for model behavior can outperform the human-derived resource-rational benchmark. Figure S7 summarizes estimated total points under six optimization methods: random, extreme, the original resource-rational optimum (Optimal–RR), and three model-tailored variants:

- **Optimal-AI** is a descriptive surrogate policy to logged optimal-task trajectories. For each observed trajectory, the ordered reveals are replayed to reconstruct the intermediate belief states, and then we fit three components conditioned on the state s: $P(\text{stop}|s)$, $P(\text{reveal cell}c|s, \text{continue})$, $P(\text{choose basket}j|s, \text{stop})$. The optimizer searches over sets of three additional revealed cells (as in the Resource Rational-optimal nudge) and chooses the set with maximal predicted meta-level return

- In **Optimal-MLP**, we simply train a direct supervised scorer neural network model from the fully observed task state. The training data for this model consist of logged optimal-task rows with known payoff matrix, weight vector, baseline random-3 reveals, observed additional three-cell nudge, and final net earnings.

- Finally, **Optimal-R3** (Resource-Rational Residual) attempts to learn a deviation from the resource-rational optimal nudge rather than a free-form strategy. in which we start from the RR three-cell set, enumerate the neighborhood of single-swap edits, score each candidate set, and then select the highest-scoring candidate.

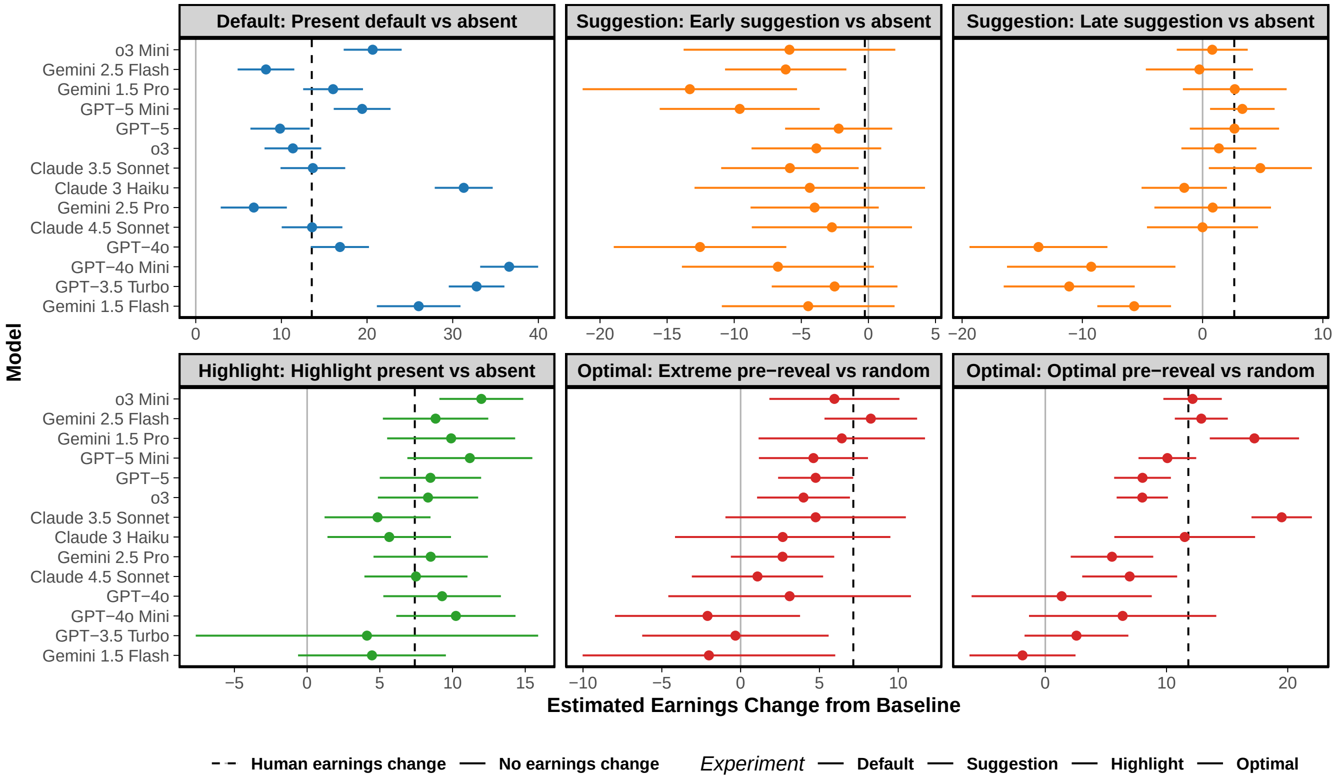

Fig. S4. Change in earnings induced by nudges relative to a no-change baseline and relative to the human treatment effect. Informative nudges can yield human-level or greater gains for stronger models, whereas weaker models often underperform the human benchmark; when nudges are not uniformly beneficial, more models fail to match the human earnings shift.

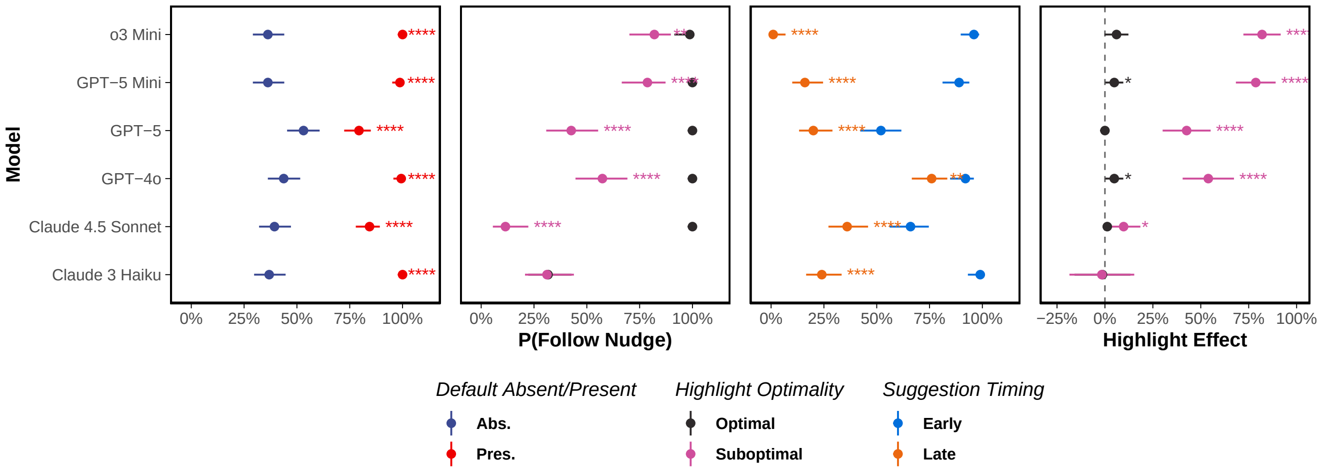

Fig. S5. Estimated marginal probabilities of following each nudge in the housing experiment. Panels show default absent/present, highlight optimality, suggestion timing, and the highlight effect relative to control (since there is no human baseline provided here).

For humans and for the stronger reasoning-oriented models, the resource-rational nudge remains the best intervention, suggesting that it already captures a robust structure of useful information. Model-specific optimization helps most for weaker or more idiosyncratic models. Thus, tailoring nudges to model behavior can sometimes increase influence or performance, but these gains do not systematically dominate the original human-derived optimal nudge.

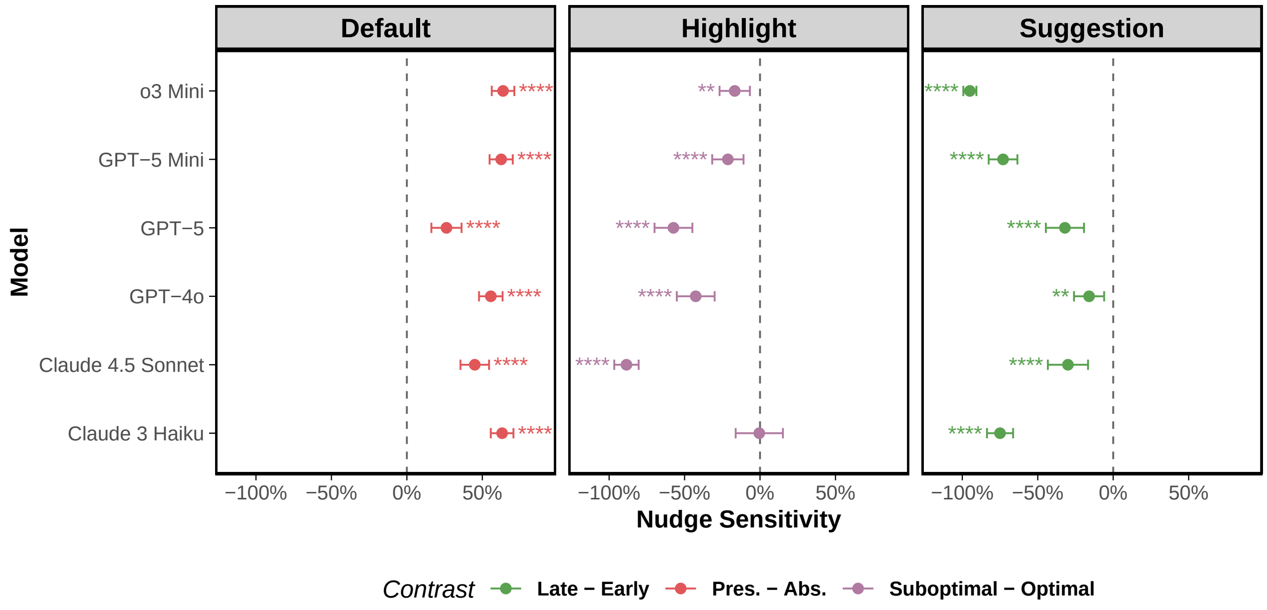

Fig. S6. Nudge-sensitivity contrasts in the housing experiment. Positive values for defaults indicate more frequent following when the default is present; negative values for highlights and suggestions indicate weaker following of suboptimal versus optimal highlights and of late versus early suggestions.

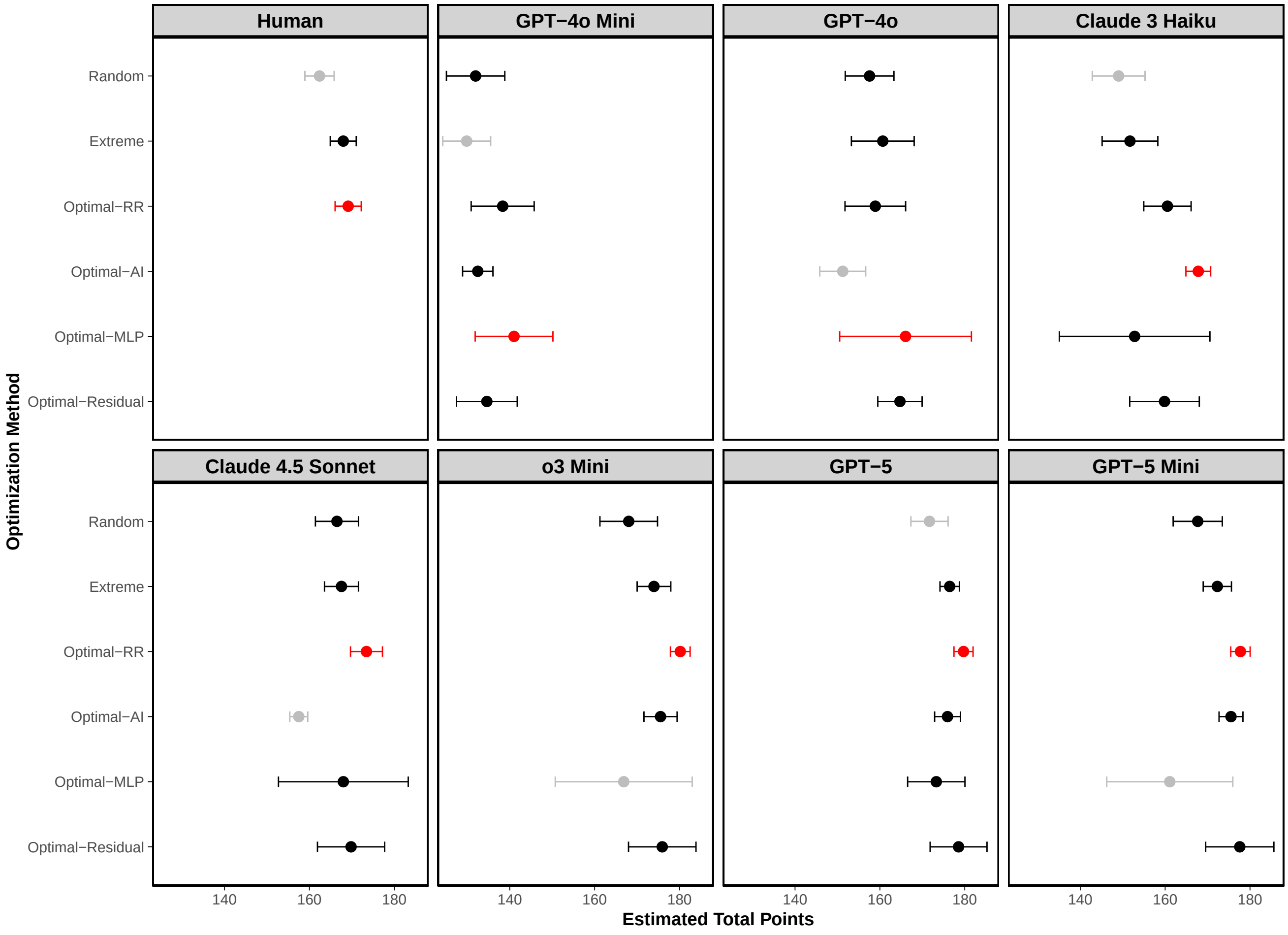

Fig. S7. Estimated total points under alternative optimization schemes. Panels correspond to models; points and intervals show estimated marginal means and 95% confidence intervals. The resource-rational optimum remains competitive for stronger models, whereas model-specific optimizers help mainly for weaker or more idiosyncratic models.

5. Heterogeneity of Nudge Sensitivity

The main text reports aggregate acceptance rates. Here we examine whether these effects vary systematically with task structure. Figure S8 breaks down default acceptance probability by grid shape (number of baskets × number of prizes). With this analysis, we seek to test whether nudge sensitivity is modulated by task complexity.

Humans show modest variation across grid shapes, with acceptance rates remaining relatively stable other than exhibiting a base rate bias (the base rate is 50% in two-basket trials, but only 20% in five-basket trials). LLMs in several cases uniformly or near-uniformly exhibit heightened sensitivity (o3 Mini, GPT-4o Mini and 4o, Claude 3 Haiku, and GPT-5 Mini, for instance). Other models exhibit more pronounced heterogeneity. For example, Claude 4.5 Sonnet, 3.5 Sonnet, and Gemini 1.5 Pro show significantly heichtened sensitivity in the 5×2 grid. It is not clear a priori what accounts for this difference, but it is unlikely to be driven by task complexity in terms of number of choices, since the 5×5 grid does not indicate a heightened sensitivity.

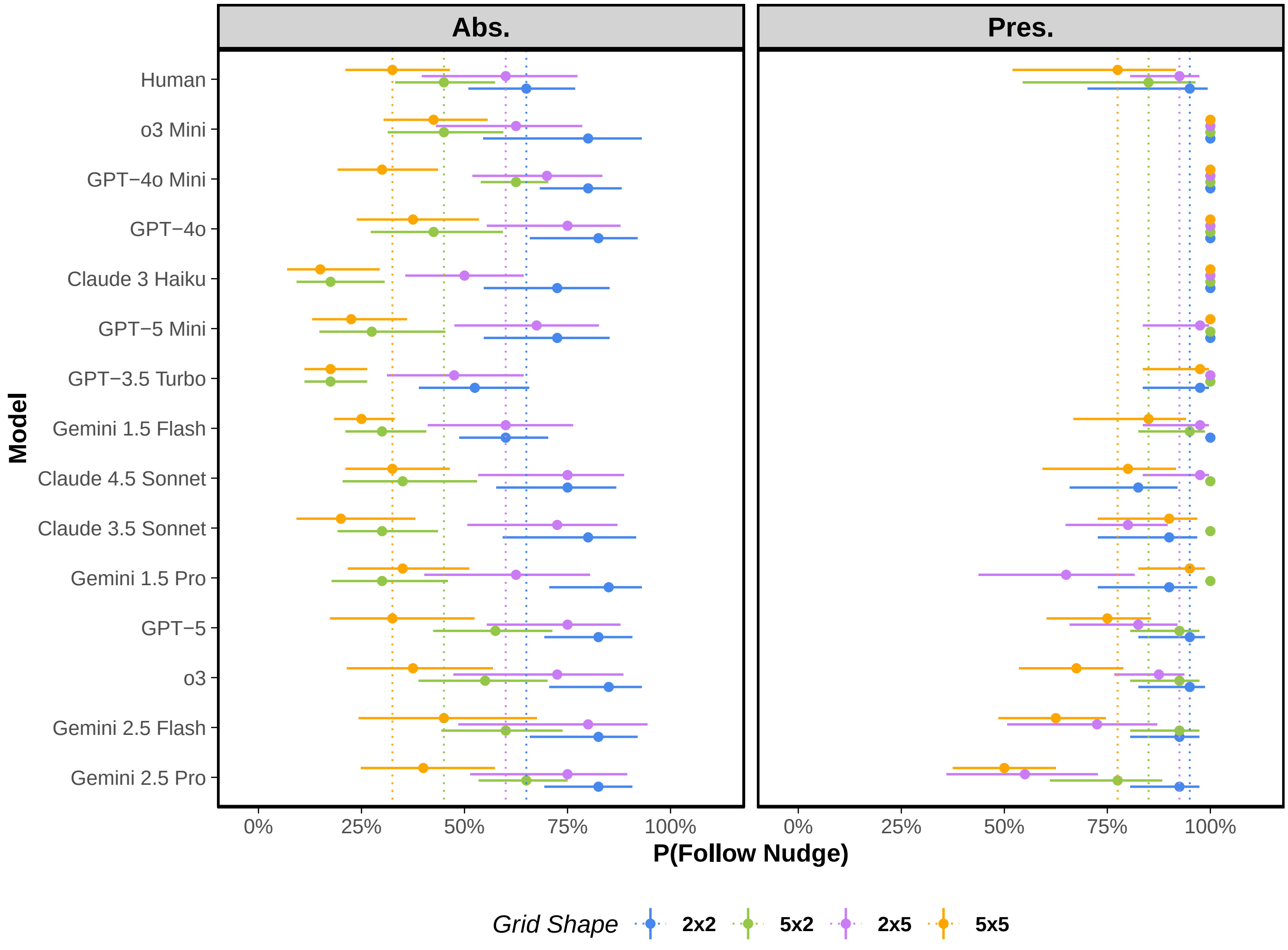

Fig. S8. Probability of choosing the default nudged basket: heterogeneity by grid shape (baskets × prizes).

We further examined how agents respond to defaults when the optimal choice is less clear. Figure S9 plots the probability of initially rejecting a default and subsequently choosing the corresponding basket anyway, as a function of "idiosyncracy"—the L1 deviation of prize weights from the uniform distribution. Lower idiosyncracy implies the default is more clearly optimal based on initial conditions (since defaults were chosen based on unweighted prize counts).

Humans appear to overall trend upwards, i.e. they slightly more frequently reject defaults in high-idiosyncracy trials where the default's optimality is more ambiguous *a priori*. Here we subset to the strongest frontier LLMs for clarity, and show that they have much higher base rates of rejecting the default and then ultimately choosing the nudged index, however they also display an appropriate sensitivity to the idiosyncracy level.

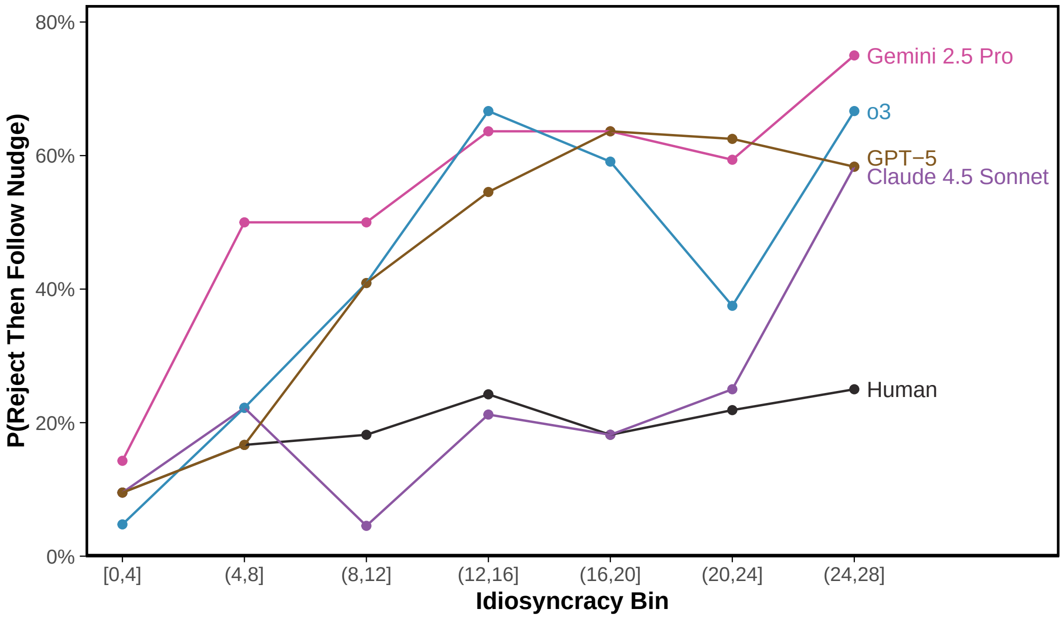

Fig. S9. Probability of initially rejecting the default nudged basket and then accepting it by idiosyncracy (L1 deviation from the uniform weight vector). At lower idiosyncracy, the default is more clearly optimal and thus should be taken more often.

We also look at heterogeneity in the Suggestion trials. Not all suggestion-following is equally consequential. Figure S10 addresses a critical question: when models switch to a suggested basket, how often is this switch *undesirable*—i.e. a switch from a higher-value to a lower-value option? We do not have switch data for the human participants, as this is not recorded by the original authors. Here, several LLMs exhibit substantial bad-switch rates, sometimes exceeding 50%, and none are immune from bad switches. This adds evidence that model nudge sensitivity is not always strategically calibrated: agents follow suggestions even when the suggested option does not improve expected outcomes.

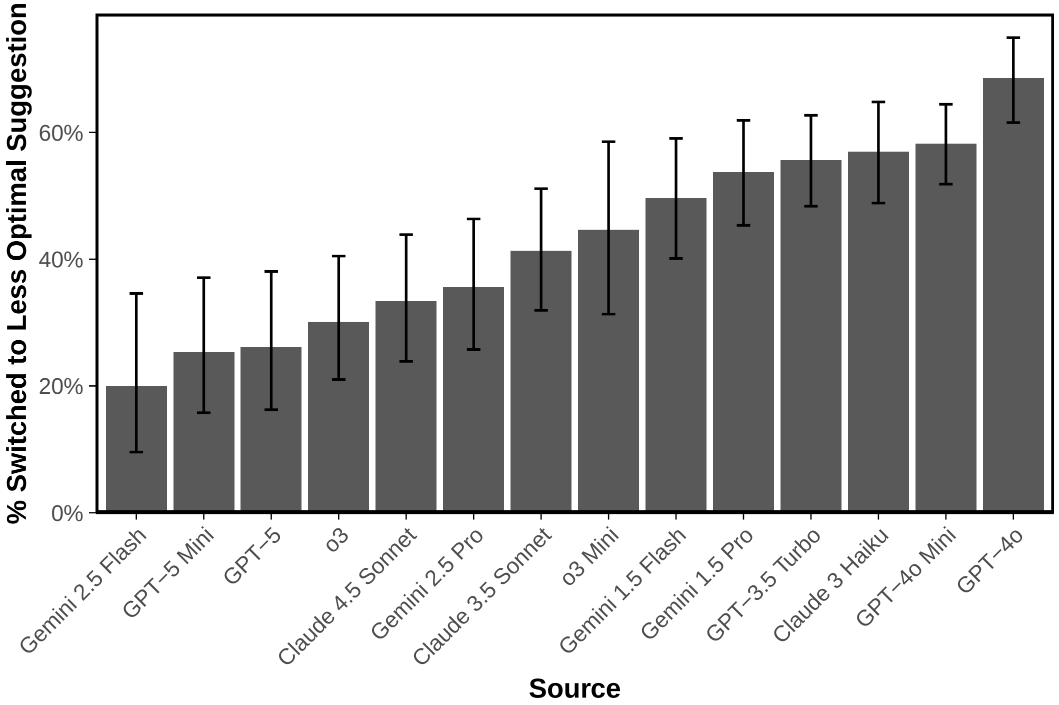

Fig. S10. When a model switches to the (late) suggested basket, how often this switch is undesirable (i.e. switching from a higher-value to a lower-value basket).

Finally, we provide Figure S11 as a supplementary analysis to the main text, where we primarily evaluated highlight sensitivity by *optimality* but did not focus on comparison to the control condition. Here, we observe that several models reveal

from the highlighted row at rates exceeding the human rate when the highlight is displayed to the model, but the few-shot data appears to mitigate this effect in some cases and bring rates closer to human rates.

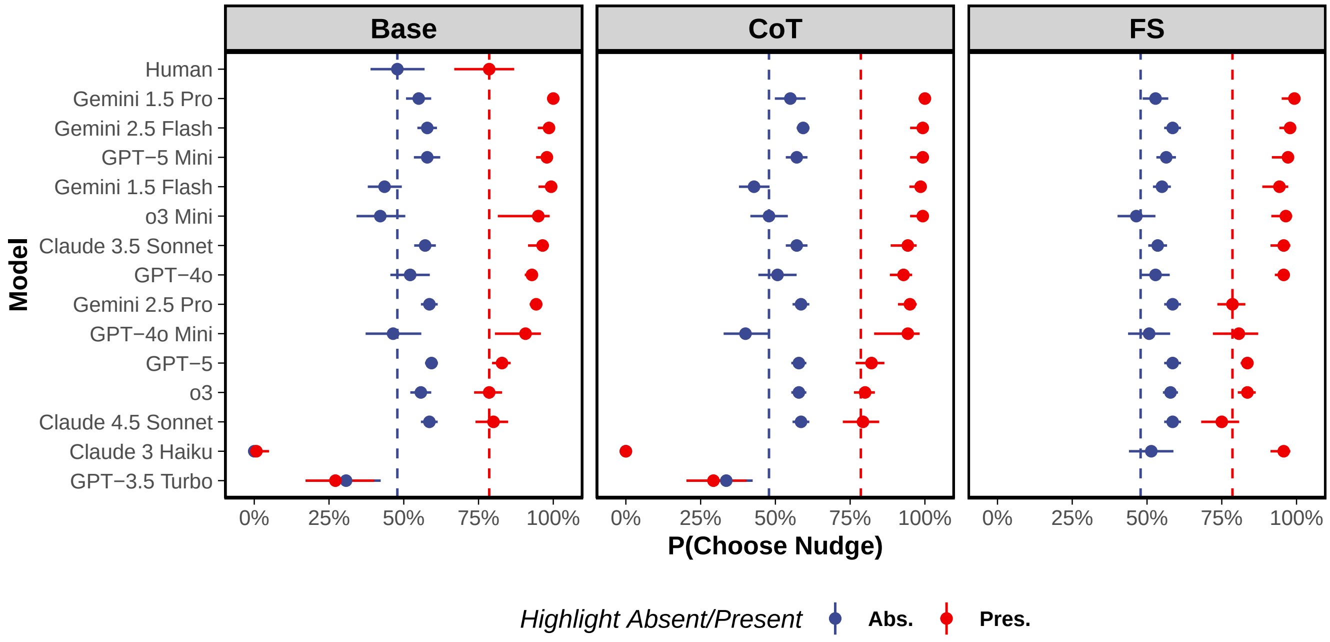

Fig. S11. Estimated marginal reveal from nudged prize rates (making the first selection from the highlighted row) for humans and models under the highlight nudge vs. the control condition.

6. Regression Tables

Tables S2 to S5 present complete linear regression tables, where the regression models predict net earnings across the four experimental conditions (defaults, suggestions, highlighting, and optimal nudges). Models include fixed effects for data source (human vs. each LLM), prompting condition (base, chain-of-thought, or few-shot), trial condition or other variables such as nudge optimality, and their interactions, with cluster-robust standard errors. As a counterpart to the earnings regression tables, we also provide estimated marginal means in Table S6, which may be more interpretable.

Table S2. Linear regression predicting net earnings in the default nudge experiment.

	(1)
(Intercept)	160.631**** (3.377)
source [GPT-3.5 Turbo]	-14.519** (3.371)
source [GPT-4o Mini]	-19.813**** (2.155)
source [GPT-4o]	2.069 (1.936)
source [Gemini 1.5 Flash]	-13.669**** (1.866)
source [Gemini 1.5 Pro]	-0.881 (1.563)
source [Gemini 2.5 Flash]	-0.413 (2.339)
source [Gemini 2.5 Pro]	4.831* (1.704)
source [Claude 3 Haiku]	-15.494** (3.282)
source [Claude 3.5 Sonnet]	1.775 (2.415)
source [Claude 4.5 Sonnet]	3.862 (2.411)
source [o3 Mini]	2.150 (2.494)
source [o3]	3.781* (1.318)
source [GPT-5]	5.669** (1.633)
source [GPT-5 Mini]	-1.144 (2.614)
trial nudge [Pres.]	14.013* (5.013)
method [CoT]	2.319 (1.862)
method [FS]	-0.894 (1.849)
source [GPT-3.5 Turbo] × trial nudge [Pres.]	18.300** (5.391)
source [GPT-4o Mini] × trial nudge [Pres.]	24.712*** (3.783)
source [GPT-4o] × trial nudge [Pres.]	2.831 (3.846)
source [Gemini 1.5 Flash] × trial nudge [Pres.]	15.962*** (2.610)
source [Gemini 1.5 Pro] × trial nudge [Pres.]	0.525 (3.319)
source [Gemini 2.5 Flash] × trial nudge [Pres.]	-5.925 (3.533)
source [Gemini 2.5 Pro] × trial nudge [Pres.]	-7.081 (3.419)
source [Claude 3 Haiku] × trial nudge [Pres.]	20.394** (5.237)
source [Claude 3.5 Sonnet] × trial nudge [Pres.]	1.831 (4.126)
source [Claude 4.5 Sonnet] × trial nudge [Pres.]	-1.219 (3.280)

source [o3 Mini] × trial nudge [Pres.]	2.750 (4.327)
source [o3] × trial nudge [Pres.]	-2.125 (2.879)
source [GPT-5] × trial nudge [Pres.]	-3.469 (3.162)
source [GPT-5 Mini] × trial nudge [Pres.]	5.881 (4.622)
source [GPT-3.5 Turbo] × method [CoT]	-2.100 (1.942)
source [GPT-4o Mini] × method [CoT]	-2.831 (2.315)
source [GPT-4o] × method [CoT]	-3.637 (2.246)
source [Gemini 1.5 Flash] × method [CoT]	-1.112 (1.620)
source [Gemini 1.5 Pro] × method [CoT]	-1.306 (2.110)
source [Gemini 2.5 Flash] × method [CoT]	-1.644 (1.437)
source [Gemini 2.5 Pro] × method [CoT]	-2.956 (2.069)
source [Claude 3 Haiku] × method [CoT]	-0.425 (2.026)
source [Claude 3.5 Sonnet] × method [CoT]	-1.631 (1.801)
source [Claude 4.5 Sonnet] × method [CoT]	-3.337 (2.491)
source [o3 Mini] × method [CoT]	-4.287 (2.200)
source [o3] × method [CoT]	-0.337 (2.004)
source [GPT-5] × method [CoT]	-0.787 (1.674)
source [GPT-4o Mini] × method [FS]	7.806* (3.233)
source [GPT-4o] × method [FS]	2.225 (1.292)
source [Gemini 1.5 Flash] × method [FS]	12.044*** (1.977)
source [Gemini 1.5 Pro] × method [FS]	-0.625 (1.065)
source [Gemini 2.5 Flash] × method [FS]	2.569 (2.369)
source [Gemini 2.5 Pro] × method [FS]	1.800 (2.010)
source [Claude 3 Haiku] × method [FS]	8.356* (2.735)
source [Claude 3.5 Sonnet] × method [FS]	5.125 (2.374)
source [Claude 4.5 Sonnet] × method [FS]	0.444 (2.396)
source [o3 Mini] × method [FS]	-8.819* (3.037)
source [o3] × method [FS]	-0.281 (2.941)
source [GPT-5] × method [FS]	2.100 (1.870)
trial nudge [Pres.] × method [CoT]	-2.456 (1.894)
trial nudge [Pres.] × method [FS]	1.056 (1.869)
(source [GPT-3.5 Turbo] × trial nudge [Pres.]) × method [CoT]	2.831 (2.195)
(source [GPT-4o Mini] × trial nudge [Pres.]) × method [CoT]	2.969 (2.238)
(source [GPT-4o] × trial nudge [Pres.]) × method [CoT]	3.775 (2.281)
(source [Gemini 1.5 Flash] × trial nudge [Pres.]) × method [CoT]	-0.319 (2.150)
(source [Gemini 1.5 Pro] × trial nudge [Pres.]) × method [CoT]	1.787 (2.237)
(source [Gemini 2.5 Flash] × trial nudge [Pres.]) × method [CoT]	1.131 (1.385)
(source [Gemini 2.5 Pro] × trial nudge [Pres.]) × method [CoT]	2.825 (1.983)
(source [Claude 3 Haiku] × trial nudge [Pres.]) × method [CoT]	0.562 (2.073)
(source [Claude 3.5 Sonnet] × trial nudge [Pres.]) × method [CoT]	1.281 (1.877)
(source [Claude 4.5 Sonnet] × trial nudge [Pres.]) × method [CoT]	3.825 (2.592)
(source [o3 Mini] × trial nudge [Pres.]) × method [CoT]	4.425 (2.221)
(source [o3] × trial nudge [Pres.]) × method [CoT]	0.050 (2.584)
(source [GPT-5] × trial nudge [Pres.]) × method [CoT]	-0.044 (1.520)
(source [GPT-4o Mini] × trial nudge [Pres.]) × method [FS]	-7.969* (3.253)
(source [GPT-4o] × trial nudge [Pres.]) × method [FS]	-2.387 (1.312)
(source [Gemini 1.5 Flash] × trial nudge [Pres.]) × method [FS]	-10.125** (2.819)
(source [Gemini 1.5 Pro] × trial nudge [Pres.]) × method [FS]	4.100** (1.229)
(source [Gemini 2.5 Flash] × trial nudge [Pres.]) × method [FS]	0.600 (2.402)
(source [Gemini 2.5 Pro] × trial nudge [Pres.]) × method [FS]	-1.887 (2.277)
(source [Claude 3 Haiku] × trial nudge [Pres.]) × method [FS]	-8.519* (2.771)
(source [Claude 3.5 Sonnet] × trial nudge [Pres.]) × method [FS]	-6.387* (2.485)
(source [Claude 4.5 Sonnet] × trial nudge [Pres.]) × method [FS]	-0.069 (2.701)
(source [o3 Mini] × trial nudge [Pres.]) × method [FS]	8.656* (3.021)
(source [o3] × trial nudge [Pres.]) × method [FS]	-0.287 (2.863)
(source [GPT-5] × trial nudge [Pres.]) × method [FS]	-0.675 (2.294)
R2	0.104
R2 Adj.	0.098
RMSE	32.01
Std.Errors	by: participant_id

Table S3. Linear regression predicting net earnings in the suggestion nudge experiment.

	(1)
(Intercept)	169.635**** (3.802)
source [GPT-3.5 Turbo]	-24.395**** (2.599)
source [GPT-4o Mini]	-27.555**** (3.724)
source [GPT-4o]	-1.245 (2.545)
source [Gemini 1.5 Flash]	-18.765*** (3.936)
source [Gemini 1.5 Pro]	-6.025 (4.442)
source [Gemini 2.5 Flash]	-0.375 (3.088)
source [Gemini 2.5 Pro]	4.805 (2.884)
source [Claude 3 Haiku]	-17.835** (4.745)
source [Claude 3.5 Sonnet]	-10.385* (3.745)
source [Claude 4.5 Sonnet]	6.285 (3.107)
source [o3 Mini]	-10.045** (3.432)
source [o3]	5.525 (2.731)

source [GPT-5]	7.735* (2.895)
source [GPT-5 Mini]	-1.265 (3.771)
trial nudge [Early]	0.465 (4.491)
trial nudge [Late]	2.206 (4.134)
method [CoT]	-2.410 (2.788)
method [FS]	6.630* (2.812)
source [GPT-3.5 Turbo] × trial nudge [Early]	0.705 (3.513)
source [GPT-4o Mini] × trial nudge [Early]	-9.665 (4.803)
source [GPT-4o] × trial nudge [Early]	-10.775* (3.876)
source [Gemini 1.5 Flash] × trial nudge [Early]	0.485 (3.988)
source [Gemini 1.5 Pro] × trial nudge [Early]	-18.065* (7.884)
source [Gemini 2.5 Flash] × trial nudge [Early]	-6.285* (2.823)
source [Gemini 2.5 Pro] × trial nudge [Early]	-4.105 (2.986)
source [Claude 3 Haiku] × trial nudge [Early]	-3.525 (6.817)
source [Claude 3.5 Sonnet] × trial nudge [Early]	-6.315 (5.327)
source [Claude 4.5 Sonnet] × trial nudge [Early]	-5.805 (3.785)
source [o3 Mini] × trial nudge [Early]	-6.305 (6.237)
source [o3] × trial nudge [Early]	-3.715 (3.075)
source [GPT-5] × trial nudge [Early]	-5.585 (3.163)
source [GPT-5 Mini] × trial nudge [Early]	-9.325* (3.850)
source [GPT-3.5 Turbo] × trial nudge [Late]	-10.516** (3.012)
source [GPT-4o Mini] × trial nudge [Late]	-13.666** (4.256)
source [GPT-4o] × trial nudge [Late]	-12.956** (3.611)
source [Gemini 1.5 Flash] × trial nudge [Late]	-7.836* (3.334)
source [Gemini 1.5 Pro] × trial nudge [Late]	3.334 (3.082)
source [Gemini 2.5 Flash] × trial nudge [Late]	-2.506 (2.952)
source [Gemini 2.5 Pro] × trial nudge [Late]	-2.036 (3.358)
source [Claude 3 Haiku] × trial nudge [Late]	-2.826 (5.479)
source [Claude 3.5 Sonnet] × trial nudge [Late]	8.294 (3.943)
source [Claude 4.5 Sonnet] × trial nudge [Late]	-1.586 (2.369)
source [o3 Mini] × trial nudge [Late]	-1.856 (4.750)
source [o3] × trial nudge [Late]	-1.306 (2.630)
source [GPT-5] × trial nudge [Late]	-0.626 (2.132)
source [GPT-5 Mini] × trial nudge [Late]	0.674 (3.281)
source [GPT-3.5 Turbo] × method [CoT]	9.740** (3.111)
source [GPT-4o Mini] × method [CoT]	5.930 (3.992)
source [GPT-4o] × method [CoT]	5.850 (4.223)
source [Gemini 1.5 Flash] × method [CoT]	2.850 (3.422)
source [Gemini 1.5 Pro] × method [CoT]	3.120 (3.776)
source [Gemini 2.5 Flash] × method [CoT]	5.340 (2.564)
source [Gemini 2.5 Pro] × method [CoT]	2.210 (2.906)
source [Claude 3 Haiku] × method [CoT]	0.530 (5.427)
source [Claude 3.5 Sonnet] × method [CoT]	4.100 (4.522)
source [Claude 4.5 Sonnet] × method [CoT]	2.930 (2.628)
source [o3 Mini] × method [CoT]	0.310 (4.933)
source [o3] × method [CoT]	1.010 (3.532)
source [GPT-5] × method [CoT]	1.810 (2.845)
source [GPT-4o Mini] × method [FS]	-6.420 (5.109)
source [GPT-4o] × method [FS]	-0.960 (4.934)
source [Gemini 1.5 Flash] × method [FS]	5.130 (3.990)
source [Gemini 1.5 Pro] × method [FS]	-8.060 (4.820)
source [Gemini 2.5 Flash] × method [FS]	-6.550 (3.907)
source [Gemini 2.5 Pro] × method [FS]	-5.170 (2.942)
source [Claude 3 Haiku] × method [FS]	-10.520* (3.746)
source [Claude 3.5 Sonnet] × method [FS]	14.190** (4.540)
source [Claude 4.5 Sonnet] × method [FS]	-9.660* (4.002)
source [o3 Mini] × method [FS]	-8.960 (4.283)
source [o3] × method [FS]	-8.160 (3.940)
source [GPT-5] × method [FS]	-10.620* (3.831)
trial nudge [Early] × method [CoT]	2.580 (3.305)
trial nudge [Late] × method [CoT]	4.570 (3.358)
trial nudge [Early] × method [FS]	-4.770 (2.985)
trial nudge [Late] × method [FS]	-3.290 (3.447)
(source [GPT-3.5 Turbo] × trial nudge [Early]) × method [CoT]	-8.880** (2.359)
(source [GPT-4o Mini] × trial nudge [Early]) × method [CoT]	-3.170 (4.968)
(source [GPT-4o] × trial nudge [Early]) × method [CoT]	-4.390 (4.729)
(source [Gemini 1.5 Flash] × trial nudge [Early]) × method [CoT]	-4.210 (3.139)
(source [Gemini 1.5 Pro] × trial nudge [Early]) × method [CoT]	-3.100 (4.580)
(source [Gemini 2.5 Flash] × trial nudge [Early]) × method [CoT]	-3.980 (3.362)
(source [Gemini 2.5 Pro] × trial nudge [Early]) × method [CoT]	-4.840 (3.236)
(source [Claude 3 Haiku] × trial nudge [Early]) × method [CoT]	-2.350 (6.192)
(source [Claude 3.5 Sonnet] × trial nudge [Early]) × method [CoT]	-3.670 (4.252)
(source [Claude 4.5 Sonnet] × trial nudge [Early]) × method [CoT]	-0.070 (3.559)
(source [o3 Mini] × trial nudge [Early]) × method [CoT]	-1.690 (5.582)
(source [o3] × trial nudge [Early]) × method [CoT]	-2.710 (4.572)
(source [GPT-5] × trial nudge [Early]) × method [CoT]	1.150 (4.277)
(source [GPT-3.5 Turbo] × trial nudge [Late]) × method [CoT]	-9.630* (4.182)
(source [GPT-4o Mini] × trial nudge [Late]) × method [CoT]	-4.290 (3.482)
(source [GPT-4o] × trial nudge [Late]) × method [CoT]	-8.040 (3.983)

(source [Gemini 1.5 Flash] × trial nudge [Late]) × method [CoT]	-2.480 (4.334)
(source [Gemini 1.5 Pro] × trial nudge [Late]) × method [CoT]	-4.400 (4.049)
(source [Gemini 2.5 Flash] × trial nudge [Late]) × method [CoT]	-7.260* (3.319)
(source [Gemini 2.5 Pro] × trial nudge [Late]) × method [CoT]	-3.240 (3.279)
(source [Claude 3 Haiku] × trial nudge [Late]) × method [CoT]	-4.610 (6.209)
(source [Claude 3.5 Sonnet] × trial nudge [Late]) × method [CoT]	-5.100 (4.021)
(source [Claude 4.5 Sonnet] × trial nudge [Late]) × method [CoT]	-7.000* (3.087)
(source [o3 Mini] × trial nudge [Late]) × method [CoT]	1.290 (6.812)
(source [o3] × trial nudge [Late]) × method [CoT]	-3.920 (4.412)
(source [GPT-5] × trial nudge [Late]) × method [CoT]	-5.380 (3.554)
(source [GPT-4o Mini] × trial nudge [Early]) × method [FS]	12.730 (7.167)
(source [GPT-4o] × trial nudge [Early]) × method [FS]	-0.130 (4.602)
(source [Gemini 1.5 Flash] × trial nudge [Early]) × method [FS]	-9.910** (3.331)
(source [Gemini 1.5 Pro] × trial nudge [Early]) × method [FS]	18.170** (6.208)
(source [Gemini 2.5 Flash] × trial nudge [Early]) × method [FS]	5.140 (3.835)
(source [Gemini 2.5 Pro] × trial nudge [Early]) × method [FS]	5.930 (3.498)
(source [Claude 3 Haiku] × trial nudge [Early]) × method [FS]	0.610 (4.435)
(source [Claude 3.5 Sonnet] × trial nudge [Early]) × method [FS]	5.860 (7.359)
(source [Claude 4.5 Sonnet] × trial nudge [Early]) × method [FS]	10.120* (4.135)
(source [o3 Mini] × trial nudge [Early]) × method [FS]	3.750 (5.365)
(source [o3] × trial nudge [Early]) × method [FS]	3.020 (4.512)
(source [GPT-5] × trial nudge [Early]) × method [FS]	9.750 (4.989)
(source [GPT-4o Mini] × trial nudge [Late]) × method [FS]	9.600 (4.796)
(source [GPT-4o] × trial nudge [Late]) × method [FS]	-1.940 (4.803)
(source [Gemini 1.5 Flash] × trial nudge [Late]) × method [FS]	1.020 (4.445)
(source [Gemini 1.5 Pro] × trial nudge [Late]) × method [FS]	-5.470 (3.657)
(source [Gemini 2.5 Flash] × trial nudge [Late]) × method [FS]	6.080 (4.211)
(source [Gemini 2.5 Pro] × trial nudge [Late]) × method [FS]	3.970 (3.324)
(source [Claude 3 Haiku] × trial nudge [Late]) × method [FS]	0.610 (5.807)
(source [Claude 3.5 Sonnet] × trial nudge [Late]) × method [FS]	-13.270* (5.881)
(source [Claude 4.5 Sonnet] × trial nudge [Late]) × method [FS]	3.840 (4.730)
(source [o3 Mini] × trial nudge [Late]) × method [FS]	-1.210 (5.447)
(source [o3] × trial nudge [Late]) × method [FS]	4.010 (5.351)
(source [GPT-5] × trial nudge [Late]) × method [FS]	7.310 (4.567)
R2	0.130
R2 Adj.	0.122
RMSE	32.23
Std.Errors	by: participant_id

Table S4. Linear regression predicting net earnings in the highlight nudge experiment.

	(1)
(Intercept)	167.371**** (6.691)
source [GPT-3.5 Turbo]	-27.564* (9.027)
source [GPT-4o Mini]	-51.286**** (4.890)
source [GPT-4o]	-9.479 (7.818)
source [Gemini 1.5 Flash]	-18.629** (4.919)
source [Gemini 1.5 Pro]	-1.064 (6.822)
source [Gemini 2.5 Flash]	0.221 (6.287)
source [Gemini 2.5 Pro]	4.993 (6.543)
source [Claude 3 Haiku]	-13.250* (5.772)
source [Claude 3.5 Sonnet]	1.614 (7.203)
source [Claude 4.5 Sonnet]	8.007 (6.600)
source [o3 Mini]	-7.179 (5.271)
source [o3]	8.471 (6.215)
source [GPT-5]	9.364 (6.709)
source [GPT-5 Mini]	0.107 (6.066)
trial nudge [Pres.]	9.564 (4.915)
method [CoT]	0.207 (2.139)
method [FS]	1.457 (2.420)
source [GPT-3.5 Turbo] × trial nudge [Pres.]	-7.386 (6.895)
source [GPT-4o Mini] × trial nudge [Pres.]	3.479 (3.527)
source [GPT-4o] × trial nudge [Pres.]	1.857 (5.350)
source [Gemini 1.5 Flash] × trial nudge [Pres.]	-5.900 (3.733)
source [Gemini 1.5 Pro] × trial nudge [Pres.]	-0.221 (3.978)
source [Gemini 2.5 Flash] × trial nudge [Pres.]	1.800 (4.416)
source [Gemini 2.5 Pro] × trial nudge [Pres.]	-2.236 (4.581)
source [Claude 3 Haiku] × trial nudge [Pres.]	-8.886 (5.930)
source [Claude 3.5 Sonnet] × trial nudge [Pres.]	-3.564 (4.903)
source [Claude 4.5 Sonnet] × trial nudge [Pres.]	-2.529 (4.747)
source [o3 Mini] × trial nudge [Pres.]	3.329 (3.573)
source [o3] × trial nudge [Pres.]	-4.293 (5.403)
source [GPT-5] × trial nudge [Pres.]	-2.293 (5.430)
source [GPT-5 Mini] × trial nudge [Pres.]	3.786 (5.224)
source [GPT-3.5 Turbo] × method [CoT]	-11.429 (7.117)
source [GPT-4o Mini] × method [CoT]	7.850 (5.552)
source [GPT-4o] × method [CoT]	2.029 (1.624)

source [Gemini 1.5 Flash] × method [CoT]	-0.207 (2.139)
source [Gemini 1.5 Pro] × method [CoT]	-4.407 (4.968)
source [Gemini 2.5 Flash] × method [CoT]	0.871 (2.307)
source [Gemini 2.5 Pro] × method [CoT]	-1.164 (2.321)
source [Claude 3 Haiku] × method [CoT]	-0.250 (4.111)
source [Claude 3.5 Sonnet] × method [CoT]	4.329 (2.889)
source [Claude 4.5 Sonnet] × method [CoT]	-0.036 (2.170)
source [o3 Mini] × method [CoT]	2.786 (3.146)
source [o3] × method [CoT]	-1.450 (1.791)
source [GPT-5] × method [CoT]	-0.100 (2.280)
source [GPT-4o Mini] × method [FS]	34.929**** (5.289)
source [GPT-4o] × method [FS]	11.664* (4.752)
source [Gemini 1.5 Flash] × method [FS]	0.907 (5.411)
source [Gemini 1.5 Pro] × method [FS]	-0.743 (4.658)
source [Gemini 2.5 Flash] × method [FS]	1.629 (2.420)
source [Gemini 2.5 Pro] × method [FS]	0.564 (2.678)
source [Claude 3 Haiku] × method [FS]	-16.107** (4.839)
source [Claude 3.5 Sonnet] × method [FS]	4.650 (2.876)
source [Claude 4.5 Sonnet] × method [FS]	-3.664 (3.937)
source [o3 Mini] × method [FS]	-3.686 (3.531)
source [o3] × method [FS]	-5.993 (3.208)
source [GPT-5] × method [FS]	-2.486 (3.051)
trial nudge [Pres.] × method [CoT]	-4.007 (3.525)
trial nudge [Pres.] × method [FS]	-2.471 (3.099)
(source [GPT-3.5 Turbo] × trial nudge [Pres.]) × method [CoT]	12.286* (4.120)
(source [GPT-4o Mini] × trial nudge [Pres.]) × method [CoT]	1.221 (4.545)
(source [GPT-4o] × trial nudge [Pres.]) × method [CoT]	3.771 (2.664)
(source [Gemini 1.5 Flash] × trial nudge [Pres.]) × method [CoT]	4.793 (3.439)
(source [Gemini 1.5 Pro] × trial nudge [Pres.]) × method [CoT]	8.664 (5.804)
(source [Gemini 2.5 Flash] × trial nudge [Pres.]) × method [CoT]	1.007 (3.803)
(source [Gemini 2.5 Pro] × trial nudge [Pres.]) × method [CoT]	4.614 (3.478)
(source [Claude 3 Haiku] × trial nudge [Pres.]) × method [CoT]	6.714 (5.014)
(source [Claude 3.5 Sonnet] × trial nudge [Pres.]) × method [CoT]	1.364 (2.908)
(source [Claude 4.5 Sonnet] × trial nudge [Pres.]) × method [CoT]	3.121 (3.846)
(source [o3 Mini] × trial nudge [Pres.]) × method [CoT]	3.900 (5.541)
(source [o3] × trial nudge [Pres.]) × method [CoT]	7.329 (3.641)
(source [GPT-5] × trial nudge [Pres.]) × method [CoT]	4.686 (3.518)
(source [GPT-4o Mini] × trial nudge [Pres.]) × method [FS]	-3.179 (7.011)
(source [GPT-4o] × trial nudge [Pres.]) × method [FS]	-3.707 (4.215)
(source [Gemini 1.5 Flash] × trial nudge [Pres.]) × method [FS]	4.071 (5.914)
(source [Gemini 1.5 Pro] × trial nudge [Pres.]) × method [FS]	-0.500 (5.774)
(source [Gemini 2.5 Flash] × trial nudge [Pres.]) × method [FS]	-2.121 (3.837)
(source [Gemini 2.5 Pro] × trial nudge [Pres.]) × method [FS]	5.371 (3.521)
(source [Claude 3 Haiku] × trial nudge [Pres.]) × method [FS]	14.671* (5.359)
(source [Claude 3.5 Sonnet] × trial nudge [Pres.]) × method [FS]	1.643 (1.903)
(source [Claude 4.5 Sonnet] × trial nudge [Pres.]) × method [FS]	4.700 (5.712)
(source [o3 Mini] × trial nudge [Pres.]) × method [FS]	-0.157 (5.237)
(source [o3] × trial nudge [Pres.]) × method [FS]	8.279 (4.850)
(source [GPT-5] × trial nudge [Pres.]) × method [FS]	5.421 (3.626)
R2	0.162
R2 Adj.	0.156
RMSE	34.76
Std.Errors	by: participant_id

Table S5. Linear regression predicting net earnings in the optimal nudge experiment.

	(1)
(Intercept)	162.680**** (3.533)
source [GPT-3.5 Turbo]	-17.510** (4.736)
source [GPT-4o Mini]	-30.740*** (4.831)
source [GPT-4o]	-5.110 (4.322)
source [Gemini 1.5 Flash]	-22.470** (4.736)
source [Gemini 1.5 Pro]	-10.300* (3.444)
source [Gemini 2.5 Flash]	-1.590 (2.675)
source [Gemini 2.5 Pro]	2.400 (3.455)
source [Claude 3 Haiku]	-13.640*** (2.455)
source [Claude 3.5 Sonnet]	-5.730 (2.727)
source [Claude 4.5 Sonnet]	3.810 (3.589)
source [o3 Mini]	5.350 (4.192)
source [o3]	7.000 (3.252)
source [GPT-5]	9.010* (3.434)
source [GPT-5 Mini]	5.000 (4.344)
nudge_type [Extreme]	7.150* (2.445)
nudge_type [Optimal]	11.800* (4.123)
source [GPT-3.5 Turbo] × nudge_type [Extreme]	-7.480 (6.192)
source [GPT-4o Mini] × nudge_type [Extreme]	-9.250 (4.185)
source [GPT-4o] × nudge_type [Extreme]	-4.040 (5.019)

source [Gemini 1.5 Flash] × nudge_type [Extreme]	-9.160 (5.229)
source [Gemini 1.5 Pro] × nudge_type [Extreme]	-0.730 (4.439)
source [Gemini 2.5 Flash] × nudge_type [Extreme]	1.110 (2.470)
source [Gemini 2.5 Pro] × nudge_type [Extreme]	-4.490 (3.401)
source [Claude 3 Haiku] × nudge_type [Extreme]	-4.480 (5.193)
source [Claude 3.5 Sonnet] × nudge_type [Extreme]	-2.390 (3.693)
source [Claude 4.5 Sonnet] × nudge_type [Extreme]	-6.080* (2.442)
source [o3 Mini] × nudge_type [Extreme]	-1.200 (4.074)
source [o3] × nudge_type [Extreme]	-3.160 (2.940)
source [GPT-5] × nudge_type [Extreme]	-2.390 (2.851)
source [GPT-5 Mini] × nudge_type [Extreme]	-2.530 (3.367)
source [GPT-3.5 Turbo] × nudge_type [Optimal]	-9.230 (5.516)
source [GPT-4o Mini] × nudge_type [Optimal]	-5.420 (5.875)
source [GPT-4o] × nudge_type [Optimal]	-10.450 (4.778)
source [Gemini 1.5 Flash] × nudge_type [Optimal]	-13.680 (6.862)
source [Gemini 1.5 Pro] × nudge_type [Optimal]	5.450 (3.528)
source [Gemini 2.5 Flash] × nudge_type [Optimal]	1.070 (2.434)
source [Gemini 2.5 Pro] × nudge_type [Optimal]	-6.300 (3.710)
source [Claude 3 Haiku] × nudge_type [Optimal]	-0.300 (3.675)
source [Claude 3.5 Sonnet] × nudge_type [Optimal]	7.700* (2.533)
source [Claude 4.5 Sonnet] × nudge_type [Optimal]	-4.840 (4.320)
source [o3 Mini] × nudge_type [Optimal]	0.350 (4.190)
source [o3] × nudge_type [Optimal]	-3.800 (3.612)
source [GPT-5] × nudge_type [Optimal]	-3.780 (3.798)
source [GPT-5 Mini] × nudge_type [Optimal]	-1.730 (4.486)
R2	0.209
R2 Adj.	0.201
RMSE	26.35
Std.Errors	by: participant_id

Table S6. Estimated marginal mean earnings (SE) across all models and conditions.

	Estimated Earnings (SE)									
source	Default / Abs.	Default / Pres.	Highlight / Abs.	Highlight / Pres.	Suggestion / Abs.	Suggestion / Early	Suggestion / Late	Optimal / Random	Optimal / Extreme	Optimal / Optimal
Human	161.1 (3.8)	174.7 (2.8)	167.9 (6.7)	175.3 (4)	171 (3.7)	170.8 (3.5)	173.7 (2.3)	162.7 (3.5)	169.8 (1.7)	174.5 (1.3)
GPT-3.5 Turbo	145.9**** (3.1)	178.7 (1.7)	136.6** (6.9)	140.7**** (6)	149.9**** (3.6)	147.4**** (2.4)	138.8**** (2.8)	145.2*** (4.2)	144.8**** (3)	147.7**** (2.2)
GPT-4o Mini	143**** (1.8)	179.5 (1.7)	130.9**** (2.6)	141.1**** (2.1)	143.3**** (3.1)	136.6**** (3.6)	134.1**** (3.6)	131.9**** (3.7)	129.8**** (3)	138.3**** (3.9)
GPT-4o	162.7 (2.1)	179.5 (1.7)	163 (2.9)	172.3 (2.1)	171.4 (3.9)	158.9** (3.3)	157.8**** (2.9)	157.6 (3)	160.7 (3.9)	158.9**** (3.8)
Gemini 1.5 Flash	151.1**** (2.1)	177.1 (2.5)	149.5* (2.6)	154*** (2.6)	154.9*** (3.7)	150.4**** (3.3)	149.2**** (1.6)	140.2**** (2.3)	138.2**** (4.1)	138.3**** (2.2)
Gemini 1.5 Pro	159.6 (2.3)	175.6 (1.8)	165.1 (2.5)	175 (2.2)	163.4 (3.8)	150.1**** (4.1)	166* (2.2)	152.4** (3.1)	158.8** (2.7)	169.6* (1.9)
Gemini 2.5 Flash	161 (2.1)	169.2 (1.7)	169 (1.9)	177.8 (1.8)	170.3 (4.3)	164.1* (2.3)	170 (2.3)	161.1 (2.4)	169.3 (1.5)	174 (1.1)
Gemini 2.5 Pro	165.6 (2.3)	172.3 (2)	172.7 (2.4)	181.2 (2)	174.9 (4.4)	170.9 (2.4)	175.7 (2.5)	165.1 (2.8)	167.7 (1.7)	170.6* (1.7)
Claude 3 Haiku	148.3*** (1.8)	179.5 (1.7)	149.2* (2.2)	154.9*** (2.2)	149.9**** (3.3)	145.5**** (4.4)	148.3**** (1.8)	149**** (3.3)	151.7**** (3.5)	160.5**** (3)
Claude 3.5 Sonnet	164 (1.9)	177.7 (1.9)	172.5 (2)	177.4 (1.9)	166.8 (2.2)	160.9** (2.6)	171.6 (2.2)	156.9 (3.5)	161.7* (2.9)	176.4 (1.3)
Claude 4.5 Sonnet	164 (2.1)	177.6 (1.8)	174.7 (2.2)	182.2 (1.8)	175.1 (4.5)	172.4 (3)	175.1 (2.4)	166.5 (2.7)	167.6 (2.1)	173.4 (2)
o3 Mini	158.9 (1.6)	179.5 (1.7)	160.4 (2.3)	172.4 (1.5)	158.1** (3.5)	152.2**** (4)	158.9**** (1.5)	168 (3.6)	174 (2.1)	180.2**** (1.2)
o3	164.7 (2.4)	176 (1.7)	173.9 (2.3)	182.2 (1.8)	174.2 (4.3)	170.3 (2.5)	175.5 (1.6)	169.7 (2.5)	173.7 (1.5)	177.7* (1.1)
GPT-5	167.2** (2.5)	177 (1.8)	176.4 (2.3)	184.9* (1.8)	175.8 (4.1)	173.6 (2)	178.5 (1.9)	171.7* (2.3)	176.4** (1.2)	179.7**** (1.2)
GPT-5 Mini	160 (1.4)	179.4 (1.7)	168 (2)	179.2 (2.2)	169.8 (3.8)	160.2** (3)	173.1 (1.4)	167.7 (3.1)	172.3 (1.8)	177.7* (1.2)

For the reasoning-augmented model experiments (with GPT-5, Claude 4.5 Sonnet, and Gemini 2.5 Pro) Tables S7 to S9 report regression tables where regression models predict the number of reasoning tokens consumed per step at different effort levels. These analyses quantify the computational cost of achieving more human-like robustness to nudges, as discussed in the main text.

Table S7. Linear regression predicting reasoning tokens per step in the default nudge experiment.

	(1)
(Intercept)	-0.000 (0.000)
model [Gemini 2.5 Pro]	-1039.944**** (55.236)
model [Claude 4.5 Sonnet]	-1058.917**** (51.322)
reasoning effort [Low]	355.424**** (16.973)
reasoning effort [Medium]	1105.664**** (52.743)
trial nudge [Pres.]	0.000 (0.000)
model [Gemini 2.5 Pro] × reasoning effort [Low]	710.427**** (52.881)
model [Claude 4.5 Sonnet] × reasoning effort [Low]	740.393**** (47.011)
model [Gemini 2.5 Pro] × trial nudge [Pres.]	254.023** (70.687)
model [Claude 4.5 Sonnet] × trial nudge [Pres.]	227.201** (62.311)
reasoning effort [Low] × trial nudge [Pres.]	-82.616** (21.640)
reasoning effort [Medium] × trial nudge [Pres.]	-98.802 (66.284)
(model [Gemini 2.5 Pro] × reasoning effort [Low]) × trial nudge [Pres.]	-113.510 (74.445)
(model [Claude 4.5 Sonnet] × reasoning effort [Low]) × trial nudge [Pres.]	-61.087 (72.627)
R2	0.757
R2 Adj.	0.756
RMSE	195.33
Std.Errors	by: participant_id

Table S8. **Linear regression predicting reasoning tokens per step in the highlight nudge experiment.**

	(1)
(Intercept)	-0.000 (0.000)
model [Gemini 2.5 Pro]	-586.280**** (13.408)
model [Claude 4.5 Sonnet]	-617.780**** (14.741)
reasoning effort [Low]	182.908**** (5.250)
reasoning effort [Medium]	651.397**** (14.720)
trial nudge [Pres.]	0.000 (0.000)
model [Gemini 2.5 Pro] × reasoning effort [Low]	416.635**** (10.699)
model [Claude 4.5 Sonnet] × reasoning effort [Low]	460.018**** (12.857)
model [Gemini 2.5 Pro] × trial nudge [Pres.]	30.435 (23.624)
model [Claude 4.5 Sonnet] × trial nudge [Pres.]	10.253 (24.294)
reasoning effort [Low] × trial nudge [Pres.]	22.933 (11.594)
reasoning effort [Medium] × trial nudge [Pres.]	-10.166 (24.452)
(model [Gemini 2.5 Pro] × reasoning effort [Low]) × trial nudge [Pres.]	-52.471* (16.359)
(model [Claude 4.5 Sonnet] × reasoning effort [Low]) × trial nudge [Pres.]	-32.532 (18.364)
R2	0.901
R2 Adj.	0.900
RMSE	71.27
Std.Errors	by: participant_id

Table S9. Linear regression predicting reasoning tokens per step in the suggestion nudge experiment.

	(1)
(Intercept)	-0.000 (0.000)
model [Gemini 2.5 Pro]	-749.722**** (25.362)
model [Claude 4.5 Sonnet]	-761.573**** (25.491)
reasoning_effort [Low]	244.562**** (13.624)
reasoning_effort [Medium]	797.538**** (25.111)
trial_nudge [Early]	0.000 (0.000)
trial_nudge [Late]	0.000 (0.000)
model [Gemini 2.5 Pro] × reasoning_effort [Low]	518.256**** (25.663)
model [Claude 4.5 Sonnet] × reasoning_effort [Low]	543.606**** (25.079)
model [Gemini 2.5 Pro] × trial_nudge [Early]	-180.232 (99.749)
model [Claude 4.5 Sonnet] × trial_nudge [Early]	-221.651* (88.372)
model [Gemini 2.5 Pro] × trial_nudge [Late]	46.012 (23.275)
model [Claude 4.5 Sonnet] × trial_nudge [Late]	-69.798* (25.218)
reasoning_effort [Low] × trial_nudge [Early]	50.176** (14.627)
reasoning_effort [Medium] × trial_nudge [Early]	246.509* (92.584)
reasoning_effort [Low] × trial_nudge [Late]	34.377 (18.350)
reasoning_effort [Medium] × trial_nudge [Late]	60.663* (26.126)
model [Gemini 2.5 Pro] × reasoning_effort [Low] × trial_nudge [Early]	148.814 (105.638)
model [Claude 4.5 Sonnet] × reasoning_effort [Low] × trial_nudge [Early]	186.745 (95.922)
model [Gemini 2.5 Pro] × reasoning_effort [Low] × trial_nudge [Late]	-70.123** (20.815)
model [Claude 4.5 Sonnet] × reasoning_effort [Low] × trial_nudge [Late]	26.857 (24.295)
R2	0.727
R2 Adj.	0.725
RMSE	185.73
Std.Errors	by: participant_id

7. Estimated Marginal Means Tables

Tables in this section provide probability-scale estimates (as *estimated marginal means* via the `emmeans` package) of nudge following rates for all models and conditions. These provide full details for the nudge sensitivity results discussed in the main text. For logistic regression models, raw coefficients are difficult to interpret; these tables transform results to the probability scale to support more direct interpretation and comparison. Note that, in all tables, † indicates perfect separation (estimate at the boundary, typically 1.0) under which p-values computed from logistic regression are not reliable.

Tables S10 to S12 provide these for the main experiments' nudge sensitivity results, Tables S13 to S15 do the same for nudge sensitivity results in the reasoning experiments.

Table S10. Estimated marginal means (SE) for the default nudge experiment.

Model	Method	Abs.	Pres.
Human	Base	0.506 (0.042)	0.875 (0.055)
GPT-3.5 Turbo	Base	0.337**** (0.017)	0.988* (0.008)
GPT-4o Mini	Base	0.606 (0.021)	1† (0)
GPT-4o	Base	0.594 (0.049)	1† (0)
Gemini 1.5 Flash	Base	0.438 (0.039)	0.944** (0.026)

Gemini 1.5 Pro	Base	0.531 (0.038)	0.875 (0.031)
Gemini 2.5 Flash	Base	0.669 (0.057)	0.8 (0.032)
Gemini 2.5 Pro	Base	0.656* (0.043)	0.687 (0.021)
Claude 3 Haiku	Base	0.387 (0.037)	1† (0)
Claude 3.5 Sonnet	Base	0.506 (0.048)	0.9 (0.021)
Claude 4.5 Sonnet	Base	0.544 (0.055)	0.9 (0.034)
o3 Mini	Base	0.575 (0.05)	1† (0)
o3	Base	0.625 (0.037)	0.856 (0.013)
GPT-5	Base	0.619 (0.032)	0.862 (0.016)
GPT-5 Mini	Base	0.475 (0.043)	0.994* (0.006)
GPT-3.5 Turbo	CoT	0.331**** (0.025)	0.994* (0.006)
GPT-4o Mini	CoT	0.594 (0.028)	1† (0)
GPT-4o	CoT	0.575 (0.046)	1† (0)
Gemini 1.5 Flash	CoT	0.45 (0.033)	0.931 (0.027)
Gemini 1.5 Pro	CoT	0.525 (0.033)	0.85 (0.028)
Gemini 2.5 Flash	CoT	0.688** (0.044)	0.769 (0.03)
Gemini 2.5 Pro	CoT	0.65* (0.039)	0.706 (0.016)
Claude 3 Haiku	CoT	0.438 (0.047)	1† (0)
Claude 3.5 Sonnet	CoT	0.506 (0.04)	0.919 (0.019)
Claude 4.5 Sonnet	CoT	0.519 (0.051)	0.894 (0.034)
o3 Mini	CoT	0.556 (0.034)	1† (0)
o3	CoT	0.638* (0.051)	0.819 (0.024)
GPT-5	CoT	0.663** (0.039)	0.838 (0.023)
GPT-5 Mini	CoT	0.494 (0.033)	0.969 (0.01)
GPT-4o Mini	FS	0.531 (0.033)	1† (0)
GPT-4o	FS	0.6 (0.042)	1† (0)
Gemini 1.5 Flash	FS	0.556 (0.032)	0.956 (0.013)
Gemini 1.5 Pro	FS	0.456 (0.034)	0.937 (0.028)
Gemini 2.5 Flash	FS	0.619 (0.047)	0.769 (0.023)
Gemini 2.5 Pro	FS	0.588 (0.052)	0.662* (0.023)
Claude 3 Haiku	FS	0.469 (0.04)	1† (0)
Claude 3.5 Sonnet	FS	0.556 (0.043)	0.825 (0.023)
Claude 4.5 Sonnet	FS	0.513 (0.044)	0.819 (0.024)
o3 Mini	FS	0.419 (0.031)	1† (0)
o3	FS	0.6 (0.039)	0.806 (0.017)
GPT-5	FS	0.581 (0.054)	0.944 (0.015)
GPT-5 Mini	FS	0.494 (0.036)	1† (0)

Table S11. Estimated marginal means (SE) for the highlight nudge experiment.

Model	Method	Optimal	Suboptimal
Human	Base	0.939 (0.061)	0.569 (0.09)
GPT-3.5 Turbo	Base	0.244*** (0.061)	0.31** (0.07)
GPT-4o Mini	Base	0.951 (0.038)	0.845* (0.056)
GPT-4o	Base	1† (0)	0.828** (0.022)
Gemini 1.5 Flash	Base	1† (0)	0.983*** (0.017)
Gemini 1.5 Pro	Base	1† (0)	1† (0)
Gemini 2.5 Flash	Base	1† (0)	0.966**** (0.022)
Gemini 2.5 Pro	Base	1† (0)	0.862*** (0.021)
Claude 3 Haiku	Base	0.012**** (0.012)	0**** (0)
Claude 3.5 Sonnet	Base	1† (0)	0.914** (0.04)
Claude 4.5 Sonnet	Base	1† (0)	0.517 (0.072)
o3 Mini	Base	0.963**** (0.036)	0.931** (0.039)
o3	Base	1† (0)	0.483 (0.065)
GPT-5	Base	1† (0)	0.586 (0.032)
GPT-5 Mini	Base	1† (0)	0.948*** (0.027)
GPT-3.5 Turbo	CoT	0.268*** (0.055)	0.328** (0.063)
GPT-4o Mini	CoT	0.976 (0.025)	0.897* (0.06)
GPT-4o	CoT	1† (0)	0.828* (0.047)
Gemini 1.5 Flash	CoT	1† (0)	0.966*** (0.023)
Gemini 1.5 Pro	CoT	1† (0)	1† (0)
Gemini 2.5 Flash	CoT	1† (0)	0.983** (0.018)
Gemini 2.5 Pro	CoT	1† (0)	0.879* (0.037)
Claude 3 Haiku	CoT	0**** (0)	0**** (0)
Claude 3.5 Sonnet	CoT	1† (0)	0.862* (0.05)
Claude 4.5 Sonnet	CoT	1† (0)	0.5 (0.083)
o3 Mini	CoT	1† (0)	0.983** (0.018)
o3	CoT	1† (0)	0.517 (0.049)
GPT-5	CoT	1† (0)	0.569 (0.046)
GPT-5 Mini	CoT	1† (0)	0.983** (0.017)
GPT-4o Mini	FS	0.927 (0.027)	0.638 (0.073)
GPT-4o	FS	1† (0)	0.897**** (0.028)
Gemini 1.5 Flash	FS	1† (0)	0.862* (0.049)
Gemini 1.5 Pro	FS	1† (0)	0.983*** (0.017)

Gemini 2.5 Flash	FS	1† (0)	0.948** (0.028)
Gemini 2.5 Pro	FS	1† (0)	0.483 (0.075)
Claude 3 Haiku	FS	1† (0)	0.897** (0.039)
Claude 3.5 Sonnet	FS	1† (0)	0.897** (0.038)
Claude 4.5 Sonnet	FS	1† (0)	0.397 (0.073)
o3 Mini	FS	0.988 (0.012)	0.931** (0.038)
o3	FS	1† (0)	0.603 (0.042)
GPT-5	FS	1† (0)	0.603 (0.029)
GPT-5 Mini	FS	1† (0)	0.931** (0.037)

Table S12. Estimated marginal means (SE) for the suggestion nudge experiment.

Model	Method	Early	Late
Human	Base	0.347 (0.043)	0.247 (0.042)
GPT-3.5 Turbo	Base	0.67**** (0.046)	0.77**** (0.042)
GPT-4o Mini	Base	0.92**** (0.038)	0.48* (0.07)
GPT-4o	Base	0.64**** (0.042)	0.61**** (0.054)
Gemini 1.5 Flash	Base	0.52 (0.06)	0.23 (0.044)
Gemini 1.5 Pro	Base	1† (0)	0.42** (0.046)
Gemini 2.5 Flash	Base	0.26 (0.053)	0.13* (0.036)
Gemini 2.5 Pro	Base	0.25 (0.055)	0.27 (0.06)
Claude 3 Haiku	Base	0.99**** (0.01)	0.69**** (0.069)
Claude 3.5 Sonnet	Base	0.92**** (0.041)	0.43 (0.083)
Claude 4.5 Sonnet	Base	0.29 (0.05)	0.26 (0.049)
o3 Mini	Base	0.95**** (0.022)	0.07*** (0.026)
o3	Base	0.42 (0.056)	0.26 (0.051)
GPT-5	Base	0.4 (0.057)	0.21 (0.045)
GPT-5 Mini	Base	0.75**** (0.047)	0.25 (0.039)
GPT-3.5 Turbo	CoT	0.72**** (0.043)	0.76**** (0.044)
GPT-4o Mini	CoT	0.88**** (0.035)	0.59*** (0.091)
GPT-4o	CoT	0.61*** (0.045)	0.6**** (0.053)
Gemini 1.5 Flash	CoT	0.59** (0.054)	0.13 (0.029)
Gemini 1.5 Pro	CoT	0.99**** (0.01)	0.37 (0.064)
Gemini 2.5 Flash	CoT	0.35 (0.055)	0.11** (0.023)
Gemini 2.5 Pro	CoT	0.28 (0.052)	0.2 (0.039)
Claude 3 Haiku	CoT	1† (0)	0.51*** (0.043)
Claude 3.5 Sonnet	CoT	0.93**** (0.033)	0.42* (0.06)
Claude 4.5 Sonnet	CoT	0.26 (0.047)	0.27 (0.046)
o3 Mini	CoT	0.95**** (0.026)	0.06* (0.026)
o3	CoT	0.44 (0.053)	0.22 (0.046)
GPT-5	CoT	0.33 (0.044)	0.16 (0.047)
GPT-5 Mini	CoT	0.72**** (0.058)	0.18 (0.046)
GPT-4o Mini	FS	0.73**** (0.039)	0.88**** (0.043)
GPT-4o	FS	0.67**** (0.06)	0.61**** (0.052)
Gemini 1.5 Flash	FS	0.73**** (0.042)	0.03*** (0.021)
Gemini 1.5 Pro	FS	0.85**** (0.03)	0.68**** (0.046)
Gemini 2.5 Flash	FS	0.47 (0.067)	0.11* (0.037)
Gemini 2.5 Pro	FS	0.22 (0.041)	0.23 (0.053)
Claude 3 Haiku	FS	0.98**** (0.013)	0.37 (0.144)
Claude 3.5 Sonnet	FS	0.36 (0.044)	0.21 (0.037)
Claude 4.5 Sonnet	FS	0.36 (0.059)	0.29 (0.056)
o3 Mini	FS	0.93**** (0.033)	0.34 (0.059)
o3	FS	0.45 (0.061)	0.24 (0.042)
GPT-5	FS	0.45 (0.063)	0.23 (0.066)
GPT-5 Mini	FS	0.68**** (0.05)	0.08* (0.035)

Table S13. Estimated marginal means (SE) for the default nudge reasoning experiment.

Model	Reasoning Effort	Abs.	Pres.
Human	Unknown	0.506 (0.042)	0.875 (0.055)
GPT-5	Minimal	0.675*** (0.036)	0.987*** (0.008)
Gemini 2.5 Pro	Low	0.637** (0.039)	0.881 (0.024)
GPT-5	Low	0.612 (0.049)	0.919 (0.031)
Claude 4.5 Sonnet	Low	0.575 (0.056)	0.662* (0.025)
Gemini 2.5 Pro	Medium	0.644** (0.036)	0.687* (0.031)
GPT-5	Medium	0.625** (0.028)	0.831 (0.023)
Claude 4.5 Sonnet	Medium	0.606 (0.049)	0.713 (0.019)

Table S14. Estimated marginal means (SE) for the highlight nudge reasoning experiment.

Model	Reasoning Effort	Optimal	Suboptimal
Human	Unknown	0.939 (0.061)	0.569 (0.09)
GPT-5	Minimal	$1^{\dagger}$ (0)	0.897^{*} (0.046)
Gemini 2.5 Pro	Low	$1^{\dagger}$ (0)	0.759 (0.053)
GPT-5	Low	$1^{\dagger}$ (0)	0.672 (0.038)
Claude 4.5 Sonnet	Low	$1^{\dagger}$ (0)	0.69 (0.046)
Gemini 2.5 Pro	Medium	$1^{\dagger}$ (0)	0.862^{*} (0.036)
GPT-5	Medium	$1^{\dagger}$ (0)	0.569 (0.034)
Claude 4.5 Sonnet	Medium	$1^{\dagger}$ (0)	0.569 (0.046)

Table S15. Estimated marginal means (SE) for the suggestion nudge reasoning experiment.

Model	Reasoning Effort	Early	Late
Human	Unknown	0.347 (0.043)	0.247 (0.042)
GPT-5	Minimal	0.64^{***} (0.061)	0.13^{*} (0.026)
Gemini 2.5 Pro	Low	0.42 (0.07)	0.27 (0.051)
GPT-5	Low	0.6^{**} (0.06)	0.13^{*} (0.029)
Claude 4.5 Sonnet	Low	0.28 (0.043)	0.27 (0.066)
Gemini 2.5 Pro	Medium	0.24 (0.049)	0.29 (0.059)
GPT-5	Medium	0.39 (0.052)	0.18 (0.05)
Claude 4.5 Sonnet	Medium	0.28 (0.035)	0.28 (0.056)

8. Average Cost of Reasoning

Tables S16 to S18 report average computational costs per decision step for reasoning models at different effort levels. These complement the token analyses by providing cost estimates in standardized units (cents, here), supporting comparison across providers with different pricing structures.

Table S16. Average output cost (in cents) per turn for the default nudge reasoning experiment.

Model	Reasoning Effort	Avg. Cost (cents) - Nudge Absent	Avg. Cost (cents) - Nudge Present
Gemini 2.5 Pro	Low	0.07	0.18
Gemini 2.5 Pro	Medium	0.15	0.46
GPT-5	Minimal	0.03	0.02
GPT-5	Low	0.74	0.57
GPT-5	Medium	2.24	2.04
Claude 4.5 Sonnet	Low	0.20	0.46
Claude 4.5 Sonnet	Medium	0.23	0.66

Table S17. Average output cost (in cents) per turn for the highlight nudge reasoning experiment.

Model	Reasoning Effort	Avg. Cost (cents) - Nudge Optimal	Avg. Cost (cents) - Nudge Suboptimal
Gemini 2.5 Pro	Low	0.05	0.04
Gemini 2.5 Pro	Medium	0.17	0.16
GPT-5	Minimal	0.03	0.03
GPT-5	Low	0.38	0.47
GPT-5	Medium	1.19	1.50
Claude 4.5 Sonnet	Low	0.17	0.17
Claude 4.5 Sonnet	Medium	0.19	0.20

Table S18. Average output cost (in cents) per turn for the suggestion nudge reasoning experiment.

Model	Reasoning Effort	Avg. Cost (cents) - Suggestion Absent	Avg. Cost (cents) - Suggestion Early	Avg. Cost (cents) - Suggestion Late
Gemini 2.5 Pro	Low	0.04	0.08	0.06
Gemini 2.5 Pro	Medium	0.11	0.25	0.33
GPT-5	Minimal	0.03	0.03	0.03
GPT-5	Low	0.52	0.61	0.58
GPT-5	Medium	1.62	2.11	1.74
Claude 4.5 Sonnet	Low	0.17	0.22	0.15
Claude 4.5 Sonnet	Medium	0.20	0.29	0.17

References

1. D Harrison Jr, DL Rubinfeld, Hedonic housing prices and the demand for clean air. *J. environmental economics management* **5**, 81–102 (1978).
